# Supplementary material for: What factors influence community wound care in the UK? A focus group study using the Theoretical Domains Framework
Source: BMJ Open. 2019 Jul 31;9(7):e024859. doi: 10.1136/bmjopen-2018-024859 (PMC6678001; doi:10.1136/bmjopen-2018-024859)
Supplement: Supplementary data [file bmjopen-2018-024859supp001.pdf]

Appendix 1: What Factors Influence Community Wound Care: Clinical Professional Focus Group Questions

Questions	Prompts for further exploration	TDF Domains
How do you decide which dressing or treatment to use for which patient?	What factors contribute and how is it/are they obtained? Please provide examples to explain answers<br>• Knowledge and skills,<br>○ Under/post grad training/regular updates<br>○ Peers/networking<br>○ Experience/Expertise<br>○ Preferences<br>○ Specialist support<br>○ Conferences/seminars<br>○ Pharmaceutical reps/fact sheets<br>• Research evidence<br>○ Reading journals<br>○ On-line search<br>○ National guidelines<br>○ Communicated via wound care specialists<br>• Patient and carers' influence<br>○ Lifestyle<br>○ Adherence<br>○ Choice/Preference<br>○ Anatomical factors /dressing suitability for foot in mobile patient	Skills Knowledge<br><br>Social professional role and identity<br><br>Behavioral Regulation<br><br>Environmental context and resources<br><br>Beliefs about capabilities<br><br>Memory, attention and decision processes<br><br>Beliefs about consequences<br><br>Motivation and goals<br><br>Emotion
Are there any environmental (organisational or resource based) factors that influence your prescribing practice?	Are the following enablers or barriers?<br>• Processes e.g. having a formulary in place?<br>○ If a formulary is in place is ordering from it mandatory?<br>• Product cost?<br>• Value for money?<br>○ Are some products worth paying more for e.g. silver/soft silicon?<br>○ What additional benefits do they provide?<br>○ How do you justify the additional cost?<br>• Product availability?<br>• Product knowledge? Why choose one product over another?r<br>• Memory (considering the number of products available)?<br>• Training? Competence?<br>• Caseload? Automony?<br>• Team support?	Behavioral Regulation<br><br>Environmental context and resources<br><br>Knowledge<br><br>Skills<br><br>Beliefs about capabilities<br><br>Memory, attention and decision processes<br><br>Beliefs about capabilities

Questions	Prompts for further exploration	TDF Domains
Do you have any influence on what products are included in your Trust formulary?	How?<br><br>Who else is involved?<br><br>If not you – who decides	Social professional role and identity<br><br>Skills<br><br>Environmental context and resources<br><br>Beliefs about capabilities<br><br>Nature of the behaviors
Have other people or situations ever caused you to change your wound care practices?	An incident? What happened?<br><br>Service reconfiguration? Why was this necessary?<br><br>A change in policy? Why was the policy changed? How was the change implemented?<br><br>A colleague? An expert in the field?<br><br>What processes are in place to share practice relating to product usage?<br><br>Do current networks adequately promote shared cared between teams and services? If not what do you think needs to be done to improve this?	Behavioral Regulation<br><br>Environmental context and resources<br><br>Nature of the behaviors<br><br>Social influences
How do you know you are/your service or your trust is making the right decisions regarding product use and service delivery?	Have you completed any audits of clinical care, clinical outcomes or service outcomes?<br>• Are prescribing practices audited? If so how frequently?<br>• Are prescribing skills audited? If so how frequently?<br>• Are prescribing skills regularly monitored/ appraised? If so how frequently?<br>• Do you have a PDP? Do attend regular personal development reviews? How often?<br>• Do you receive regular updates at trust or service level regarding service delivery achievements?<br>What other measures are in place to monitor prescribing practices?	Behavioral Regulation<br><br>Motivation and goals

Now you have had time to look at the wound care product expenditure……….

Questions	Prompts for further exploration	TDF Domains
Are the overall figures what you were expecting?	Is the overall spend higher or lower than you were expecting?<br><br>What do you think has caused the difference (if any)?<br><br>How do you feel about the differences or similarities with neighboring Trusts and national figures?	Knowledge<br><br>Beliefs about consequences<br><br>Behavioral Regulation<br><br>Environmental context and resources
Does the expenditure for any particular product group surprise you?	Are you surprised that it is low or high expenditure?<br><br>Why does it surprise you?<br><br>What do you think has caused this?<br><br>How do you feel about the differences with neighboring Trusts and national figures?	Knowledge<br><br>Beliefs about consequences<br><br>Environmental context and resources<br><br>Behavioral Regulation
Do you think there is over or under use of any product group?	Which group(s)? Is this over or under used?<br><br>What do you think has caused this?<br><br>Prior to seeing the figures, which product groups did you believe were used the most frequently?<br><br>Prior to seeing the figures, which product groups did you believe were used the least frequently?<br><br>How do you feel about the comparison with neighboring Trusts and national figures?	Knowledge<br><br>Skills<br><br>Beliefs about consequences<br><br>Environmental context and resources
