# Supplementary material for: What factors influence community wound care in the UK? A focus group study using the Theoretical Domains Framework
Source: BMJ Open. 2019 Jul 31;9(7):e024859. doi: 10.1136/bmjopen-2018-024859 (PMC6678001; doi:10.1136/bmjopen-2018-024859)
Supplement: Supplementary data [file bmjopen-2018-024859supp002.pdf]

Appendix 2. What Factors Influence Community Wound Care: Non-clinical Professional Focus Group Questions

Introductory statement to clarify the focus: As you all represent different organisations and have different roles, our questions will aim to establish the procurement processes and practices across four community healthcare organisations (that have partnered with CLAHRC GM to deliver this work) and neighbouring partner CCGs. When we say 'local' we are referring to the 14 services involved. We will use prompts to explore each question further so that we have a clear understanding of each of the services involved.

Questions	Prompts for further exploration	TDF Domains
What procurement processes are in place locally?	Stock list only? Prescribing only? Combination?<br><br>How is information about the systems/practices circulated to community and primary care staff? Are communication channels monitored?<br><br>Do Trusts/CCGs have a formulary?<br>• How are they compiled/updated?<br>• Who is involved?<br>• Who influences what is included?<br><br>What are the drivers for changing processes/practice? Do you have examples?<br>• Incidents?<br>• Audit findings?<br>• Service reconfiguration?<br>• Research evidence?<br>• Lessons learnt from other services?	Environmental context and resources<br><br>Knowledge<br><br>Skills<br><br>Social professional role and identity<br><br>Social influences

Questions	Prompts for further exploration	TDF Domains
Are there any factors that influence procurement decisions?	Are the following enablers or barriers?<br>• Product cost?<br>• Product availability?<br>• Product knowledge?<br>• Memory (considering the number of products available)?<br>• Training?<br>• Competence?<br>• Team support?<br>• Company reps<br>○ access monitored/unmonitored<br>○ Incentives	Behavioral Regulation<br><br>Environmental context and resources<br><br>Knowledge<br><br>Skills<br><br>Beliefs about capabilities<br><br>Memory, attention and decision processes
What locally agreed CQUINs/policies are in place for wound care management?	What do these entail?<br><br>Are there any requirements to conduct audits to monitor adherence to agreed wound care management pathway?<br>• Six monthly/yearly?<br>• What are the consequences for poor adherence<br><br>Do they include incentive schemes?<br>Does they include a commitments to reduce spend e.g. reduce silver dressings spend.<br>Do these include a commitment to undertake an ongoing programme of educational training to community or primary care staff on agreed wound management pathway (inc formulary)?<br><br>Do they include a commitment to benchmark with other organisations? (Which?)	Behavioral Regulation<br><br>Environmental context and resources<br><br>Beliefs about consequences<br><br>Motivation and goals<br>Skills
Do healthcare professionals follow policy when ordering /prescribing wound care products?	What percentage of products are ordered/prescribed 'off formulary'<br><br>Are there specific products that are prescribed regularly 'off formulary'?<br><br>Are there any incentives to promote good prescribing/ordering practices?<br><br>If a stock list exists, are stock products sometimes prescribed rather than ordered from the stock list?<br><br>What are the consequences of not following policy/ordering off formulary?<br><br>What do you think can be done to improve adherence to policy?	Behavioral Regulation<br><br>Environmental context and resources<br><br>Beliefs about consequences

The University of Manchester — NHS National Institute for Health Research

Now you have had time to look at the wound care product expenditure………..		
**Questions**	**Prompts for further exploration**	**TDF Domains**
How do you feel about the differences or similarities in expenditure locally compared to the national spend?	Is the overall spend higher or lower than you would like it to be?<br><br>How do you feel about the differences or similarities with the rest of GM and the national figures?<br><br>If overall spend is higher than average what do you think can be done to reduce the spend?	Knowledge<br><br>Beliefs about consequences<br><br>Behavioral Regulation<br><br>Environmental context and resources
Is the expenditure for any particular product higher or lower than you think it should be?	Why do to think expenditure is higher or lower?<br><br>Which type of HCP is contributing to the high/low expenditure?<br><br>How does this compares with the rest of the region and the national figures?	Knowledge<br><br>Beliefs about consequences<br><br>Environmental context and resources<br><br>Behavioral Regulation
Do you think there is over or under use of any product group?	Which group(s)? Is this over or under use?<br><br>What do you think has caused this?<br><br>How does this compare with regional and the national figures?	Knowledge<br><br>Skills<br><br>Beliefs about consequences<br><br>Environmental context and resources
