# Supplementary material for: What factors influence community wound care in the UK? A focus group study using the Theoretical Domains Framework
Source: BMJ Open. 2019 Jul 31;9(7):e024859. doi: 10.1136/bmjopen-2018-024859 (PMC6678001; doi:10.1136/bmjopen-2018-024859)
Supplement: Author's manuscript [file bmjopen-2018-024859.draft_revisions.pdf]

BMJ Open

BMJ Open is committed to open peer review. As part of this commitment we make the peer review history of every article we publish publicly available.

When an article is published we post the peer reviewers' comments and the authors' responses online. We also post the versions of the paper that were used during peer review. These are the versions that the peer review comments apply to.

The versions of the paper that follow are the versions that were submitted during the peer review process. They are not the versions of record or the final published versions. They should not be cited or distributed as the published version of this manuscript.

BMJ Open is an open access journal and the full, final, typeset and author-corrected version of record of the manuscript is available on our site with no access controls, subscription charges or pay-per-view fees (http://bmjopen.bmj.com).

If you have any questions on BMJ Open's open peer review process please email info.bmjopen@bmj.com

BMJ Open

What factors influence community wound care? A focus group study using the Theoretical Domains Framework

Journal:	*BMJ Open*
Manuscript ID	bmjopen-2018-024859
Article Type:	Research
Date Submitted by the Author:	16-Jul-2018
Complete List of Authors:	Gray, Trish; University of Manchester, Division of Nursing, Midwifery and Social Work, School of Health Sciences, Faculty of Biology, Medicine and Health<br>Wilson, Paul; University of Manchester, Alliance Manchester Business School<br>Dumville, Jo C.; University of Manchester, Division of Nursing, Midwifery and Social Work, School of Health Sciences, Faculty of Biology, Medicine and Health<br>Cullum, Nicky; The University of Manchester, Division of Nursing, Midwifery and Social Work, School of Health Sciences, Faculty of Biology, Medicine and Health
Keywords:	QUALITATIVE RESEARCH, FOCUS GROUP, WOUND MANAGEMENT, HEALTHCARE PROFESSIONAL, HEALTHCARE QUALITY, THEORETICAL DOMAINS FRAMEWORK

SCHOLARONE™
Manuscripts

What factors influence community wound care? A focus group study using the Theoretical Domains Framework

Corresponding Author

Trish Gray
Division of Nursing, Midwifery and Social Work; School of Health Sciences;
Faculty of Biology, Medicine and Health.
The University of Manchester, Room 6.332, Jean McFarlane Building,
Oxford Road, Manchester, M13 9PL.
Email: Trish.Gray@manchester.ac.uk
Tel: (+44) (0)161 306 7849

Authors:

Trish A Gray,[1,3.] Paul Wilson,[2,3] Jo C Dumville,[1,3] Nicky A Cullum.[1,3,4]

Affiliations

1 Division of Nursing, Midwifery and Social Work, School of Health Sciences, Faculty of Biology, Medicine and Health, University of Manchester, Manchester, UK

2 Alliance Manchester Business School, University of Manchester, Manchester, UK

3 NIHR CLAHRC Greater Manchester 3rd Floor Mayo Building, Salford Royal NHS Foundation Trust, Stott Lane, Salford, Manchester, UK

4 Research and Innovation Division, Central Manchester University Hospitals NHS Foundation Trust, Grafton Street, Manchester, UK

For peer review only

Word Count

Abstract 270
Main text: 4959

KEY WORDS

Qualitative research, focus group, wound management, healthcare professional, healthcare quality, Theoretical Domains Framework.

For peer review only - http://bmjopen.bmj.com/site/about/guidelines.xhtml

ABSTRACT:

Objectives: Research has found unwarranted variation in wound care across community services in the North of England, with underuse of evidence-based practice and overuse of interventions where there is little or no known patient benefit. This study explored the factors that influence community wound care to develop a deeper understanding of this variation at the patient, the service and the organisation level.

Design: Qualitative focus group study using The Theoretical Domains Framework to structure the questions, prompts and analyses.

Setting: Community healthcare settings in the North of England, UK.

Participants: Forty-six healthcare professionals who cared for patients with complex wounds and 8 medicines management personnel who were responsible for procuring wound care products participated across six focus group interviews.

Results: We found practice to be mainly based on experiential knowledge and personal preference and highly influenced by colleagues, patients and the pharmaceutical industry though not by research evidence. Factors such as incompatible information systems and financial pressures were perceived as having a negative effect on the continuity of care, patient access to wound care services, caseload and staff morale.

Conclusions: Our study provides new insight into the role that experiential learning and social influences play in determining wound care and on the limited influence of research. Participative collaboration between university and healthcare organisations may offer a route to more positive, inquisitive and resolute commitment to research use amongst healthcare professionals. Workforce pressures and limited resources are perceived to impede care by reducing patient access to services, the ability of provide holistic care as well as affecting staff morale. Organisations need to develop strategies to apportion resources wisely and redeploy skills.

For peer review only

Article Summary

Strengths and Limitations of this study

- This focus group study is the first to explore the factors that influence the management and prescribing practices in community wound care whilst seeking to explore the reasons for known variation in practice and service delivery.
- Our study provides new insight into the role experiential learning and social influences play in determining management and treatment choices and on the limited influence of evidence obtained from research.
- The focus group design stimulated discussion and through prompts we probed further giving participants the opportunity to explore their own and others' views and experiences.
- The sample was taken from community healthcare organisations in the North of England, inclusion of participants from a larger geographical population may have provided different views

For peer review only

MAIN TEXT

Introduction

In the UK, the management of people with complex wounds (wounds healing by secondary intention, such as foot, leg and pressure ulcers, open trauma and surgical wounds,[1 2] is mainly carried out by nurses in community settings with support from specialist teams (such as tissue viability, burns, vascular and dermatology). Podiatrists also play a vital role, often working as part of a shared service.

As part of a wider programme of wound care research funded by the National Institute for Health Research Collaboration for Leadership in Applied Health Research and Care (NIHR CLAHRC) Greater Manchester, we previously assessed the current management of wounds across five community healthcare organisations in the North of England.[3] The study revealed underuse of evidence-based investigations such as Doppler-aided measurement of ankle brachial pressure index (ABPI) for patients with a diagnosed venous leg ulcer.[4] We also

For peer review only - http://bmjopen.bmj.com/site/about/guidelines.xhtml

found underuse of high compression therapy, particularly two-layer compression stockings which have been found to be cost-effective relative to high compression bandages for people with venous leg ulcers.[5] Our study also exposed a potential overuse of interventions such as antimicrobial dressings where there is little or no known patient benefit.[6]

This study aimed to investigate the factors that influence wound care management and prescribing practices at the patient, the service and the organisation level with the goal of explaining variations in practice.

Methods

Design

We conducted six focus group interviews to explore the factors that influence wound management and related prescribing practices. The Theoretical Domains Framework (TDF) was used to structure the questions, prompts and analyses.[7 8] The TDF provides a theoretical lens through which to view cognitive, affective, social and environmental influences on behaviour.[9] It has been used extensively across a range of clinical areas.[10-12] Its constructs are grouped into 14 discrete domains as presented in Table 1.[7]

For peer review only

For peer review only - http://bmjopen.bmj.com/site/about/guidelines.xhtml

Table 1: The Theoretical Domains Framework domains, definitions and constructs

Domain	Definition and Constructs
Knowledge	An awareness of the existence of something [knowledge including knowledge of condition/scientific rationale, procedural knowledge, knowledge of task environment].
Skills	An ability or proficiency acquired through practice [skills, skills development, competence, ability, interpersonal skills, practice, skill assessment].
Social/Professional role and identity	A coherent set of behaviors and displayed personal qualities of an individual in a social or work setting [professional identity, professional role, social identity, identity professional boundaries, professional confidence, group identity, leadership organisational commitment].
Beliefs about capabilities	Acceptance of the truth, reality, or validity about an ability, talent, or facility that a person can put to constructive use [self-confidence, perceived competence self-efficacy, perceived behavioral control beliefs, self-esteem, empowerment professional confidence].
Optimism	The confidence that things will happen for the best or that desired goals will be attained [optimism, pessimism, unrealistic optimism, identity].
Beliefs about consequences	Acceptance of the truth, reality, or validity about outcomes of a behavior in a given situation [beliefs, outcome expectancies, characteristics of outcome expectancies anticipated regret consequences].
Reinforcement	Increasing the probability of a response by arranging a dependent relationship, or contingency, between the response and a given stimulus [rewards (proximal/distal, valued/not values, probable/improbable), incentives, punishment, consequents, reinforcement, contingencies, sanctions].
Intentions	A conscious decision to perform a behaviour or a resolve to act in a certain way [stability of intentions, stages of change model, transtheoretical model and stages of change].
Goals	Mental representations of outcomes or end states that an individual wants to achieve [goals (distal/proximal), goal priority, goal target setting, goals (autonomous/controlled) action planning, implementation intention]
Memory, attention and decision processes	The ability to retain information, focus selectively on aspects of the environment and choose between two or more alternatives [memory, attention, attention control, decision making, cognitive overload/tiredness].
Environmental context and resources	Any circumstance of a person's situation or environment that discourages or encourages the development of skills and abilities, independence, social competence and adaptive behavior [environmental stressors resources/material resources organizational culture/climate salient events/critical incidents, person, environment, interaction barriers and facilitators].
Social influences	Those interpersonal processes that can cause individuals to change their thoughts, feelings, or behaviors [social pressure, social norms, group conformity, social comparisons, group norms, social support, power intergroup conflict, alienation, group identity, modeling].
Emotion	A complex reaction pattern, involving experiential, behavioral, and psychological elements, by which individual attempts to deal with a personally significant matter or event [fear, anxiety, affect, stress, depression, positive/negative effect, burn-out].
Behavioral regulation	Anything aimed at managing or changing objectively observed or measured actions [self-monitoring, breaking habit, action planning]

© Cane et al.; licensee BioMed Central Ltd. 1900

Participants and settings

Purposive sampling was used to ensure that we recruited participants with relevant community clinical, management and procurement experience. The focus group interviews were arranged by professional group and location. Five groups were drawn from provider organisations in one defined geographical area with a sixth

For peer review only - http://bmjopen.bmj.com/site/about/guidelines.xhtml

conducted in a different geographical area but similar urban conurbation in the North of England; chosen for its well-established links with university researchers as a comparison to the other organisations where collaborative partnerships with university researchers were in their infancy. There were five focus group interviews for healthcare professionals; one for each participating healthcare provider. Healthcare professionals were eligible to participate if they regularly cared for patients with complex wounds, were wound care specialists or managed wound care services. Groups comprised of: specialist nurses (such as tissue viability or burns), podiatrists, generalist community nurses, clinical nurse managers and wound research nurses. A separate focus group was held for personnel involved in the procurement of wound care products. This group comprised: pharmacists, procurement leads, medicines management technicians, and clinical advisors to healthcare organisations. Potential participants were identified through contacts developed as part of the NIHR CLAHRC GM wound care programme and were approached via email, telephone or face-to-face meeting. We aimed to recruit 50-60 participants in total across the six groups (8 to 10 people per group), based on recommendations from existing literature.[13] An additional 20% were invited to allow for non-attendance.[14]

Data collection

The format was similar for all focus group interviews; they were facilitated by a lead (TG) with one or two co-facilitators (PW and JD). All facilitators were experienced researchers and familiar with the evidence base for wound care products. A fourth member of the research team took field notes. Before the session began, participants were asked to complete a brief demographic questionnaire that asked about years since professional registration and wound care experience; these data were used to summarise the participants involved and were not linked to particular responses or quotes. Each session was audio recorded and recordings were deleted following verification of anonymised transcripts.

Procedure

The discussion explored specific behaviours of the TDF domains and reactions to site-specific, regional and national procurement data using the questions and prompts outlined in Appendix 1. Healthcare professionals

For peer review only - http://bmjopen.bmj.com/site/about/guidelines.xhtml

were encouraged to think about factors that enable or hinder their wound care management, relating their answers to their own experiences. Through prompts we probed further, allowing participants' reactions to unfold, giving them the opportunity to explore their own and others' views. The focus group interview for procurement personnel followed an identical format with the questions more related to procurement systems and procedures (Appendix 2).

Ethical considerations

Ethics approval was sought and granted from the University of Manchester Research Ethics Committee (Refs 15272, 15327 and 2017-0559-1767) and HRA approval was sought and granted (Refs IRAS 174691, 184865 and 219918).

Patient and Public Involvement

The NIHR CLAHRC Greater Manchester Wounds Research PPI Forum provided views on their experiences with healthcare professionals and wound care services. Specialist nurses, clinical managers and a procurement lead were involved in piloting the interview schedules, after which minor amendments were made. Member validation was performed at the end of each focus group interview and following analysis; the lead facilitator provided a verbal summary taken from the field notes and a written summary was circulated via email. Participants were invited to react to the accuracy and completeness of findings. No corrections were requested.

Data Analysis

Quantitative data were stored in SPSS (IBM version 22). Demographic variables are expressed in frequencies, means and standard deviations where distributions are normal, and medians and range when skewed.

Qualitative analysis followed a seven-step process in line with the framework method:[15 16]

1. Recordings were transcribed verbatim using established methods for focus group transcription.[17]

For peer review only - http://bmjopen.bmj.com/site/about/guidelines.xhtml

2. A comprehensive familiarisation process was conducted by one researcher (TG) in which the entire data set was reviewed to contextualise the material. This began with proofreading and cross-checking against the recordings for accuracy and de-identification. Transcriptions were annotated with additional field notes and stored in NVivo 11.
3. Transcripts were then coded inductively by TG to establish the main themes.
4. An analytical framework was developed by two researchers (TG and PW). The thematic codes generated inductively were aligned with the 14 theoretical domains of the TDF.
5. Both researchers then undertook deductive coding independently, to apportion themes to domains, adding subdomains as required to ensure that important data were not omitted. Regular meetings facilitated critical exploration of participant responses and agreement on domain definitions in the context of the data set.
6. A framework matrix was developed to reduce and organise data into themes, cases and sets for ease of comparison.
7. Finally, data interpretation ensued; comparing similarities and differences between participants' views (depending on factors such as their role, experience and organisational processes) and mapping connections between categories to explore relationships and/or causality.[15]

Findings

Participant characteristics

Sixty participants were invited to attend one of six focus group interviews (mean duration: 106 minutes). Fifty-four participants attended whilst nine invited participants could not attend due to other clinical commitments or annual leave (three of whom nominated colleagues to attend in their place). Participant characteristics are presented in Table 2.

For peer review only - http://bmjopen.bmj.com/site/about/guidelines.xhtml

Table 2. Participant demographic details

Participant Demographics (n=54)	
Characteristics	***N (%)***
Gender	
Male	7 (13)
Female	47 (87)
Role group	
Specialist nurse	10 (19)
Community nurse	25 (46)
Research nurse	1 (2)
Clinical Manager	3 (5)
Podiatrist	7 (13)
Procurement personnel	8 (15)
Highest Academic Qualification	
MSc	6 (11)
BSc/BA (Hons)	27 (50)
PG Diploma	11 (20)
PG Certificate	2 (4)
Vocational Qualification	5 (9)
A level	3 (6)
Years in current role	**Mean (SD)**
Healthcare professionals	8.6 (7.4)
Procurement personnel	4.7 (4.3)
Years wounds care/procurement experience	
Healthcare professionals	14.5 (8.8)
Procurement personnel	5.7 (6.4)
Attended wound care update in last 12 months (n=54)	***N (%)***
Yes	16 (30)
No	38 (70)

Key themes identified within relevant domains

Four major themes emerged from inductive coding: *communication*, *knowledge and skills*, *variation (in care, products and services)* and *patient centred care*. In the process of mapping the emerging themes to the TDF domains, five domains dominated: *Environmental context and resources*, *Knowledge, Skills, Social influences* and *Behaviour regulation*. The domains of knowledge and skills were closely linked and frequently overlapped, therefore, we combined these.

For peer review only

For peer review only - http://bmjopen.bmj.com/site/about/guidelines.xhtml

Environmental context and resources

Delivery of care

Many participants highlighted the extensive challenges of delivering wound care due to environmental resource issues affecting staffing levels, clinic availability and information technology.

Participants across all focus group interviews expressed feeling the pressure of increased workloads. Some participants said they were working more intensely and without breaks, constantly feeling anxious that they may have missed something as time was limited between patient visits or clinic appointments. The added pressure was affecting staff morale.

"You haven't got the same skill base any more. We haven't got the same expertise, we're losing our experienced link nurse this week, and we haven't really got anybody with that level of skill in wounds to take her place........we've got 30 vacancies at the moment that haven't been filled……… you haven't got as wide a pool of knowledge and skills, have you?" (Clinical manager)

Whilst specialist clinics were being cut, service demand was increasing. Participants reported that patients previously seen in dedicated leg ulcer clinics by nurses with specialist knowledge, were now visited at home by what were suggested to be understaffed generalist community nursing teams.

"Physically running the clinic was based on when there was about six or seven [leg ulcer specialist] staff ... when it was a leg ulcer service. There's only two of us so we haven't got the capacity to cover those let alone do all the home visits." (Community nurse).

Another concern that caused unnecessary stress and treatment delays was the poor referral information received from hospital medical and nursing staff for patients discharged to the care of community nurses. Minimal information was provided about the type of wound, location, symptoms and treatment required.

For peer review only - http://bmjopen.bmj.com/site/about/guidelines.xhtml

"You constantly are ringing because they'll [ward staff] *put* [on the referral] *"care of wound", but …… what wound have they got? What operation have they had? What would you like me to do with it? It's very, very poor."* (Community nurse)

Variation in care, products and services

There was variation in the patterns of care delivery, for example whilst the majority of community healthcare organisations were cutting specialist leg ulcer clinics one had recently realigned leg ulcer care to a specialist team, which a participant reported had been found (through audit) to be an efficient model in terms of cost and clinical care. In one organisation, practice nurses (based in a general practitioner practice providing primary care for a local population) managed mobile patients with wounds, whilst community nurses cared for housebound patients with complex wounds and more complex health needs. This changed model of service delivery was felt by community nurses to have eased their workload. Within the remaining organisations, community nurses reported that they were more frequently visiting mobile patients at home to provide wound care due to the discontinuation of specialist wound clinics.

Three community services had amalgamated with hospital (acute) services in the last five years and this was reported to have had a profound effect on staff morale. Many participants felt that resourcing prioritised acute services at the expense of the community service. Participants repeatedly made reference to the differences between resources available in acute care that were limited or unavailable in the community; this included wound care products and digital technology.

"I just don't feel the acute side has got a grip at all on community services in terms of what we do…I mean, I do a specialist [acute] *clinic on a Tuesday morning and have access to all sorts of dressings. And I come back into the community….and we're very limited, we've got one foam* [dressing] *that we can use*." (Podiatrist).

Participants viewed access to photographic equipment as a valuable resource that allowed images of wounds to be sent to a podiatrist or specialist nurse for rapid diagnosis and care planning, however, only healthcare

For peer review only - http://bmjopen.bmj.com/site/about/guidelines.xhtml

professionals with access to hospital photographic equipment could make use of this service. One specialist nurse apologised for using photographic equipment that the community nurses within her organisation did not have access to.

"I do [take photographs of wounds]. I've got a camera. Sorry. It is downloaded onto a programme at the hospital. So that's probably why [I have access to it]." (Specialist nurse).

Participants noted the wide variety of information systems (electronic and paper) in use across acute, community and primary care services. This affected the speed and quality of the referral process between services as well as creating duplication of patient information due to the inability of information systems to share information.

The approach to the procurement of wound care products varied; two healthcare provider organisations obtained all products via prescription, two used a combination of prescribing and stock purchase and one operated a total stock purchase system. All trusts had wound care formularies (a list of recommended products), locally developed and updated approximately every two years by specialist nurses however the products listed varied across trusts. Some included a large range of products per product type whereas others were more restrictive. It was expected that only products listed on the formulary could be purchased or prescribed. For the organisations operating stock purchasing this was achievable as only products on the stock list could be ordered unless an off-formulary purchase (i.e. a product not listed on the formulary), was requested and sanctioned by a service lead. For organisations operating prescription only, however, formularies acted as a guide only as all wound care products were available to prescribers. One organisation had a very restrictive formulary and monitored use closely; participants found this restrictive formulary helpful in guiding and assisting decisions on product choice.

"I think it's an enabler, … there are so many [dressings to choose from] *you can go completely for something that costs so much and something that wouldn't be right … but having that formulary means that we know what*

For peer review only - http://bmjopen.bmj.com/site/about/guidelines.xhtml

we can choose and there's everything that we would really need …. , so you wouldn't need to go off it [prescribe off-formulary], *really." (Community nurse).*

Knowledge and skills

Education and training

All nurse participants agreed that there was a limited amount of wound care education for student nurses and that most wound care knowledge and skills were gained through community placements rather than in the classroom. In contrast, podiatrists received regular undergraduate wound care education.

In terms of training for post-qualified nurses, university-based wound care courses were available but these were mainly accessed by nurses preparing for a specialist wound care role and were not often attended by generalist nurses, resulting in a limited number of nursing staff with a higher level of wound care knowledge. Participants viewed wound care knowledge across other services to be particularly poor especially amongst hospital and nursing home-based nurses and GPs. Specialist nurses reported that they offered in-house wound care training for generalist community and hospital nurses but due to workforce pressures courses were often cancelled or attendance was poor. Due to these difficulties, specialist nurses relied on the pharmaceutical industry to provide wound product training sessions particularly for the launch of formulary updates. Specialist wound care nurses reported that this industry-led training was delivered in "controlled" situations to ensure pharmaceutical representatives did not promote 'off-formulary' products however, concerns were raised by many participants that the educational contributions of industry representatives were highly likely to favour their own products. Despite these concerns it was felt to be very difficult to oversee their behaviour.

With the lack of regular structured (pre and post qualification) wound care education across community nursing services, wound care knowledge appeared to be acquired through experiential learning, support from more experienced colleagues, specialist teams, podiatrists and the pharmaceutical industry.

For peer review only - http://bmjopen.bmj.com/site/about/guidelines.xhtml

Use of research evidence

Only those participants from the healthcare organisation with a history of collaborative wound care research indicated that they actively sought to keep up to date with research. Specialist nurses from this organisation talked about their established links with university researchers and their involvement in co-producing wounds research with academics. They discussed disseminating relevant research findings through electronic newsletters, workshops and meetings with community staff. Where capacity allowed, staff were supported to implement research findings. This organisation was highly research active in the wound care field; healthcare professionals had participated in research that found compression stockings to be more cost-effective than compression bandages for people with venous leg ulcers[5] and they subsequently implemented the findings into practice. Across staff from the remaining healthcare organisations, research was viewed with caution; participants reported that the dearth of good quality wound care research as reported by national guidelines[6 18 19] gave them less of an incentive to regularly search for reliable research when they felt they were unlikely to find any.

Social influences

The importance of good teamwork was frequently emphasised and acknowledged. Much of the sharing of experiences was conducted informally; advice from peers would be sought in relation to dealing with difficult wounds to address uncertainty on how to proceed. Team support appeared to alleviate some of the current workload pressures. Shared care between teams (podiatry, specialist nurse and community nursing) was prominent across four provider organisations and viewed as a valuable method for joint decision making. Only one community service had wound care link nurses whose role was to cascade new information, new research evidence and product updates from tissue viability nurses to their colleagues. Whilst the role of the link nurse was viewed as important, it had never been evaluated to measure its true potential. Capacity issues were also affecting the scope of this role.

For peer review only - http://bmjopen.bmj.com/site/about/guidelines.xhtml

As referred to above, the pharmaceutical industry is also influential; providing training and education around product use, although this varied across organisations depending on the capacity of specialist nurses to limit access and monitor sessions. Participants in procurement roles were particularly and negatively vociferous about the influence of pharmaceutical representatives yet viewed the role of policing any promotional activity as a specialist nurse responsibility.

Participants were very aware of the influence that patients have on wound care management, which at times caused difficulty finding a suitable dressing that met patients' expectations. Participants reported that some patients did not follow advice and removed dressings earlier than necessary if minor staining appeared. Participants were mindful that careful assessment and monitoring patients' adherence to therapy was necessary when making product choices. Participants also found that patients searched for information on the internet in an attempt to influence product decisions and several participants remarked that patients had requested honey-based antimicrobial dressings as they viewed honey to be a 'natural' substance.

Behaviour regulation

Dressing overuse

Monitoring prescribing or ordering behaviour appeared to be the role of specialist nurse leads and procurement personnel; the latter would flag off-formulary prescribing to specialist nurses for them to take action. When each organisation's expenditure on dressing types was discussed, community nurses and podiatrists were surprised to find that their use of antimicrobial dressings was higher than they had expected. Many reported that antimicrobial dressings (particularly silver-impregnated dressings) were used for individual patients for a two-week trial period only and then reviewed, however, they acknowledged that if use was not closely monitored there was potential for misuse. Procurement personnel and specialist nurses were aware of the high expenditure on antimicrobial dressings but acknowledged difficulties in monitoring effectively and providing adequate training and support due to capacity issues.

For peer review only - http://bmjopen.bmj.com/site/about/guidelines.xhtml

"Silver spend is still a problem and it's a long-term……I think there's still habitual use, district nurses having the time to stop and think and review and stop a treatment rather than continue." (Specialist nurse)

Specialist nurses reported that general practitioners regularly prescribed high cost antimicrobial dressings for nursing home residents. The prescription for these dressings would often be repeated without review unless the resident was referred to a specialist nurse. There was an opinion amongst participants that some prescribing of silver dressings may be accidental because dressings are listed alphabetically in some prescribing platforms and silver dressings appear first (as they are denoted by the chemical symbol for silver, 'Ag').

Standardisation

In order to standardise and better regulate prescribing behaviour across several provider organisations including primary care, plans were in progress to produce a regional formulary. However, procurement personnel were sceptical that agreement could be reached across so many organisations.

"*Trying to get large trusts to agree….trying to get ten clinicians [to agree] is pretty hard…..when you are trying to roll this out into the wider community I can only imagine it becomes ever more complex, because everyone's got an opinion*." (Procurement Lead).

Audit

Participants did report that they adhered to national targets and regulations, though there were significant concerns regarding the poor data collection capabilities of community IT systems. As the majority of community staff were still working with paper records, they found data collection for monitoring activities extremely resource intensive. For this reason, only obligatory monitoring appeared to be conducted.

Discussion

We believe this is the first study to explore factors influencing management and prescribing practices in community wound care whilst seeking to understand the reasons for known variation in practice and service

For peer review only - http://bmjopen.bmj.com/site/about/guidelines.xhtml

delivery. Overall, participants described a challenging working environment, with influences such as incompatible information systems, workforce shortages and diminishing treatment resources having a marked effect on continuity of care, patient access to services, workload and staff morale. Prescribing practices seemed to be predominantly based on experiential knowledge, personnel preference and to be highly influenced by colleagues, patients and the pharmaceutical industry.

Workforce pressures and diminishing resources

Wound care services were described by participants as a working environment characterised by increasing time pressures and diminishing resources. Clinic sessions had been cut, resulting in an increase of home visits for non-housebound patients, roles were perceived as becoming task orientated which was felt to dilute the quality of care. Participants reported that morale within their service was low; there was a rise in sickness, colleagues were leaving for less pressured roles and vacancies were not being filled. The UK has fewer nurses relative to the population than many EU countries.[20] The number of community nurses is falling, with an estimated vacancy rate of 9.4%.[21] Forty percent of experienced nurse positions (Band 6 and above) are vacant,[22] therefore, the majority of staff currently providing physical and emotional care for older people are low paid and have few qualifications.[23] Community nurses are having to work longer hours and more intensely to protect patient care which is leading to high levels of stress, low morale and increasing absence due to sickness.[24-26] Championing flexible career pathways with clear progression from entry to doctoral studies and beyond[27] and valuing the role of the healthcare assistant by identifying opportunities for learning and development[28] may improve staff recruitment. The development of integrated teams and the introduction of combined hospital and community posts to standardise practice, improve care coordination and vary work experiences could have a positive effect on retention rates.[22 29 30] Improved information technology allowing more effective time management and facilitating shared care between services, including remote consultations, may increase capacity for specialist teams allowing a greater number of patients to receive input by highly skilled healthcare professionals.

For peer review only - http://bmjopen.bmj.com/site/about/guidelines.xhtml

Incompatible information technology systems

Healthcare information technology has advanced over the past 20 years, bringing a number of different forms of electronic patient records (EPRs) into existence,[31] yet the ability to share electronic information between healthcare organisations is still relatively rare.[32 33] In this study, participants voiced frustration at the multiple electronic and paper-based systems in use, often in the same organisation, with a detrimental effect on care continuity and outcomes. EPRs that allow sharing of clinical information between healthcare providers have many advantages over paper records including: improved continuity of care due to better communication between services, reduction in time spent recording clinical information, increased legibility of records, fewer documentation errors and improved data collection for audit and research purposes.[32 34 35] Whilst there are ethical, security, cost and maintenance issues to consider, shared EPRs have the potential to improve the quality, safety, efficiency and effectiveness of healthcare and healthcare delivery systems.[36 37] Implementation of such a system requires organisational change, it appeared however, within participants' organisations that new information systems were being introduced within services on a micro-level in the absence of an organisation-wide implementation strategy.

Education and training

All nursing participants agreed that there was a lack of wound care education in basic nurse education. Wound care skills were learnt during community but not hospital placements. This was verified by participants' reference to insufficient information from hospital nursing and medical staff on referral forms and via telephone calls which cause delays in assessment and frustration for community nurses. Whilst all specialist nurse teams offered on-going wound care training to qualified nurses and healthcare assistants, cancellation or poor attendance frequently occurred due to staff shortages. By contrast wound care education was felt to be strong before and after qualification.

For peer review only

For peer review only - http://bmjopen.bmj.com/site/about/guidelines.xhtml

In the light of the current workforce issues and the difficulties community nurses had updating their wound care knowledge, other strong influences played a significant role in wound care choices such as personal, colleague and patient preferences as well as the influence of pharmaceutical company representatives. This influence can drive variation in dressing and treatment choice depending on the amount of access pharmaceutical representatives have to healthcare settings and healthcare professionals' attitude to the information they provide.[38] The ongoing cuts to continued professional development funding in the UK since 2015 may lead to greater dependence on the pharmaceutical industry for training and 'education' which is problematic due to companies' vested interests in the use of specific products.[39 40] More inter-professional education is necessary to break down professional boundaries and provide opportunities for mutual learning and joint solutions across professional groups and specialties.[41] Further investment into evaluated training interventions that are of high quality and independent is warranted to ensure education is consistent and effective; providing healthcare professionals with the confidence to make the right decisions to improve continuity and quality of care.[28 42-44]

Overuse of wound care products with little or no known patient benefit

There is a plethora of wound products available for use but, as several Cochrane systematic reviews have shown, there is a paucity of research evidence showing that products are clinically effective.[6 45-50] Despite this, product use and expenditure grow; particularly antimicrobial dressing use, where no compelling evidence or guideline recommendations exist to support routine use (Hussey et al., 2018 manuscript in draft).

We found that a restrictive formulary was viewed as enabling better patient management, particularly if guidelines accompanied the formulary. Community nurses found a formulary and guidance gave them more assurance that they were making the right decisions and specialist nurses found formularies reduced inappropriate product choices and assisted in standardising product use across their service. For the majority of organisations, however, the formulary acted as guidance only and 'off-formulary' prescribing could occur without restriction unless resources were available to monitor prescribing behaviour closely. National

For peer review only - http://bmjopen.bmj.com/site/about/guidelines.xhtml

guidelines exist to guide the use of specific products,[6 18 19 51] however, national standards to guide choice across the range of wound care products would reduce variation of product use and guide more rational prescribing.[52]

The influence of research on wound care management

Research was raised as a factor influencing wound care in only one, highly research active provider organisation. In this site, well-established links with university researchers had been highly influential. Current evidence suggests that there is an association between the engagement of individuals and healthcare organisations in research and improvements in healthcare performance.[53] In the other sites, where collaborative links with university researchers were more newly established, research informed decision making was more limited and research generally was viewed with caution. Much of the discussion around acquiring knowledge and skills to inform wound care decisions was related to experiential influences; day-to-day wound care experience, watching others and consulting with more experienced colleagues and specialists. This finding is in line with other research showing that experiential learning and the social influence of peers rather than research knowledge are major influencers on nursing practices.[54-56]

If evidence obtained from research is to inform management and practice, robust, long-term strategies to support and facilitate its use will be required. In England, the NIHR funded research that incentivises co-production of research e.g., NIHR CLAHRCs represent an on-going nationwide experiment to close the distance between research production and research use.

Limitations

We were able to recruit 8-10 participants into each group as planned but work pressures dictated the range of healthcare professionals and for one healthcare professional focus group interview there were no podiatrists which may have reduced the diversity of views, attitudes and beliefs. However there was good representation from podiatrists across the other groups ranging from senior management to junior positions. Podiatrists' utterances were coded as equally as other participants as we prompted all participants to respond to comments

For peer review only - http://bmjopen.bmj.com/site/about/guidelines.xhtml

or questions if not spontaneously offered. We feel, therefore, that podiatrists' views have been incorporated adequately. The sample was taken from community healthcare organisations in the North of England, inclusion of participants from a larger geographical population may have provided different views, however, we captured many of the issues affecting healthcare (such as work pressures, staff shortages and limited resources) across the UK[25 57 58] and further afield[59 60] due to the financial healthcare crisis worldwide.

A challenge of using the TDF was the overlap across domains such as K*nowledge* and *Skills*, *Beliefs about consequences* and *Social/professional role and identity.* Other authors have reported similar challenges.[12 61] We found coding to be heavily orientated around the *Environmental context and resources* and it took several iterations and meetings between coders (TG and PW) to ensure that our coding had not overlooked other domains. This process was assisted by the coders being experienced researchers from different professional backgrounds with one (PW) having an in-depth knowledge of the TDF. The recently published guide to using the TDF, addresses these and other challenges to promote the use of the TDF to a wider audience.[9]

Conclusions

Our study provides new insight into the role experiential learning and social influences play in determining management and treatment choices and on the limited influence of evidence obtained from research. Co-production of research evidence through participative collaboration between university and healthcare organisations may offer a route to more positive, inquisitive and resolute commitment to research amongst healthcare professionals. Workforce pressures and limited resources are perceived to impede care by reducing patient access to services, the ability of provide holistic care as well as affecting staff morale.

References

1. Cullum N, Buckley H, Dumville J, et al. Wounds Research for Patient Benefit: a 5 year programme of research. *Programme Grants Appl Res* 2016;4(13)
2. Vowden K. Complex wound or complex patient? Strategies for treatment. *Br J Community Nurs* 2005;Suppl:S6, S8, S10 passim. [published Online First: 2005/06/10]

For peer review only - http://bmjopen.bmj.com/site/about/guidelines.xhtml

3. Gray TA, Rhodes S, Atkinson RA, et al. Opportunities for better value wound care: a multiservice, cross-sectional survey of complex wounds and their care in a UK community population. *BMJ Open* 2018;8(3):e019440. doi: 10.1136/bmjopen-2017-019440 [published Online First: 2018/03/25]
4. SIGN. Management of chronic venous leg ulcers: A national clinical quideline (120): Scottish Intercollegiate Guidelines Network, 2010.
5. Ashby RL, Gabe R, Ali S, et al. Clinical and cost-effectiveness of compression hosiery versus compression bandages in treatment of venous leg ulcers (Venous leg Ulcer Study IV, VenUS IV): a randomised controlled trial. *Lancet* 2014;383(9920):871-9. doi: 10.1016/S0140-6736(13)62368-5
6. NICE. Chronic wounds: advanced wound dressings and antimicrobial dressings: Evidence summary [ESMPB2]. London: National Institute for Health and Care Excellence, 2016.
7. Cane J, O'Connor D, Michie S. Validation of the theoretical domains framework for use in behaviour change and implementation research. *Implement Sci* 2012;7:37. doi: 10.1186/1748-5908-7-37 [published Online First: 2012/04/26]
8. Michie S, Johnston M, Abraham C, et al. Making psychological theory useful for implementing evidence based practice: a consensus approach. *Qual Saf Health Care* 2005;14(1):26-33. doi: 10.1136/qshc.2004.011155 [published Online First: 2005/02/05]
9. Atkins L, Francis J, Islam R, et al. A guide to using the Theoretical Domains Framework of behaviour change to investigate implementation problems. *Implement Sci* 2017;12(1):77. doi: 10.1186/s13012-017-0605-9
10. Craig LE, Taylor N, Grimley R, et al. Development of a theory-informed implementation intervention to improve the triage, treatment and transfer of stroke patients in emergency departments using the Theoretical Domains Framework (TDF): the T(3) Trial. *Implement Sci* 2017;12(1):88. doi: 10.1186/s13012-017-0616-6
11. Murphy K, O'Connor DA, Browning CJ, et al. Understanding diagnosis and management of dementia and guideline implementation in general practice: a qualitative study using the theoretical domains framework. *Implement Sci* 2014;9:31. doi: 10.1186/1748-5908-9-31
12. Phillips CJ, Marshall AP, Chaves NJ, et al. Experiences of using the Theoretical Domains Framework across diverse clinical environments: a qualitative study. *Journal of multidisciplinary healthcare* 2015;8:139-46. doi: 10.2147/JMDH.S78458 [published Online First: 2015/04/04]
13. Krueger RA, Casey MA. Focus Groups: a practical guide for applied research, 5th edn Thousand Oaks, C.A.: Sage Publications Inc 2015.
14. Halcomb EJ, Gholizadeh L, DiGiacomo M, et al. Literature review: considerations in undertaking focus group research with culturally and linguistically diverse groups. *J Clin Nurs* 2007;16(6):1000-11. doi: 10.1111/j.1365-2702.2006.01760.x
15. Gale NK, Heath G, Cameron E, et al. Using the framework method for the analysis of qualitative data in multi-disciplinary health research. *BMC Med Res Methodol* 2013;13:117. doi: 10.1186/1471-2288-13-117
16. Ritchie J, Spencer L. Qualitative data analysis for applied policy research. In: Bryman B, Burgess R, eds. Analyzing qualitative data. London and New York: Routledge 1994:173-94.
17. Bloor M, Frankland J, Thomas M, et al. Focus Groups in Social Research. London: Sage 2001.
18. NICE. Pressure ulcers: prevention and management Clinical guideline [CG179]: National Institute for Health and Care Excellence, 2014.
19. NICE. Diabetic foot problems: prevention and management [NG19]: National Institute for Health and Care Excellence, 2015.
20. Buchan J, Charlesworth A, Gershlick B, et al. Rising pressure: the NHS workforce challenge: Workforce profile and trends of the NHS in England. London: The Health Foundation 2017.
21. OECD. The Nursing Workforce Health Statistics: Organisation for Economic Co-operation and Development 2017.
22. NHS England. Framework for commissioning community nursing. London: NHS England, 2015.
23. Cornwell J. The care of frail older people with complex needs: time for a revolution: The Sir Roger Bannister Health Sunnit, Leeds Castle. London: The King's Fund, 2012.
24. Ball J, Philippou J, Pike G, et al. Survey of district and community nurses in 2013: report to the Royal College of Nursing. London: Royal College of Nursing, 2014.
25. Maybin J, Charles A, Honeyman m. Understanding quality in district nursing services: Learning from patients, carers and staff. London: The King's Fund, 2016.
26. NQB. Safe, sustainable and productive staffing; An improvement resource for the district nursing service 1ed. London: National Quality Board 2017.
27. Public Health England. Facing the Facts, Shaping the Future: A draft health and care workforce strategy for England to 2027. London: Public Health England, 2017.
28. HEE. Raising the Bar. Shape of Caring: A Review of the Future Education and Training of Registered Nurses and Care Assistants. London: Health Education England, 2018.

For peer review only

For peer review only - http://bmjopen.bmj.com/site/about/guidelines.xhtml

29. Goodwin N, Sonala L, Thiel V, et al. Co-ordinated care for people with complex chronic conditions: Key lessons and markers for success. London: The King's Fund, 2013.
30. NICE. Transition between inpatient hospital settings and community or care home settings for adults with social care needs Nice guideline [NG27] National Institute for Health and Clinical Excellence 2015.
31. WHO. Electronic Health Records Manual for Developing Countries: World Health Organisation 2006.
32. Hoerbst A, Ammenwerth E. Electronic health records. A systematic review on quality requirements. *Methods Inf Med* 2010;49(4):320-36. doi: 10.3414/ME10-01-0038
33. Kralewski JE, Zink T, Boyle R. Factors influencing electronic clinical information exchange in small medical group practices. *J Rural Health* 2012;28(1):28-33. doi: 10.1111/j.1748-0361.2011.00372.x
34. Adane K, Muluye D, Abebe M. Processing medical data: a systematic review. *Arch Public Health* 2013;71(1):27. doi: 10.1186/0778-7367-71-27
35. Hillestad R, Bigelow J, Bower A, et al. Can electronic medical record systems transform health care? Potential health benefits, savings, and costs. *Health Aff (Millwood)* 2005;24(5):1103-17. doi: 10.1377/hlthaff.24.5.1103
36. Ozair FF, Jamshed N, Sharma A, et al. Ethical issues in electronic health records: A general overview. *Perspect Clin Res* 2015;6(2):73-6. doi: 10.4103/2229-3485.153997
37. Rezaeibagha F, Win KT, Susilo W. A systematic literature review on security and privacy of electronic health record systems: technical perspectives. *Health Inf Manag* 2015;44(3):23-38.
38. Madden M. Alienating evidence based medicine vs. innovative medical device marketing: a report on the evidence debate at a Wounds conference. *Soc Sci Med* 2012;74(12):2046-52. doi: 10.1016/j.socscimed.2012.02.026
39. Greatbatch. A false economy: Cuts to Continuing Professional Development funding for nursing, midwifery and the Allied Health Professions in England London: Council of Deans of Health, 2016.
40. Ousey K. Reducing funding for CPD courses: what does it mean for tissue viability? . *Wounds UK* 2016;13(1):10-15.
41. Frenk J, Chen L, Bhutta ZA, et al. Health professionals for a new century: transforming education to strengthen health systems in an interdependent world. *Lancet* 2010;376(9756):1923-58. doi: 10.1016/S0140-6736(10)61854-5 [published Online First: 2010/11/30]
42. Rafferty AM, Xyrichis A, Caldwell C. Post-graduate education and career pathways in nursing: a policy brief London: King's College London., 2015.
43. Thompson C, Stapley S. Do educational interventions improve nurses' clinical decision making and judgement? A systematic review. *Int J Nurs Stud* 2011;48(7):881-93. doi: 10.1016/j.ijnurstu.2010.12.005
44. Werdin F, Tennenhaus M, Schaller HE, et al. Evidence-based management strategies for treatment of chronic wounds. *Eplasty* 2009;9:e19.
45. Dumville JC, Deshpande S, O'Meara S, et al. Foam dressings for healing diabetic foot ulcers. *Cochrane Database Syst Rev* 2013(6):CD009111. doi: 10.1002/14651858.CD009111.pub3
46. Dumville JC, O'Meara S, Deshpande S, et al. Hydrogel dressings for healing diabetic foot ulcers. *Cochrane Database Syst Rev* 2013(7):CD009101. doi: 10.1002/14651858.CD009101.pub3
47. Dumville JC, Stubbs N, Keogh SJ, et al. Hydrogel dressings for treating pressure ulcers. *Cochrane Database Syst Rev* 2015(2):CD011226. doi: 10.1002/14651858.CD011226.pub2
48. Forster R, Pagnamenta F. Dressings and topical agents for arterial leg ulcers. *Cochrane Database Syst Rev* 2015(6):CD001836. doi: 10.1002/14651858.CD001836.pub3
49. Jull AB, Cullum N, Dumville JC, et al. Honey as a topical treatment for wounds. *Cochrane Database Syst Rev* 2015(3):CD005083. doi: 10.1002/14651858.CD005083.pub4
50. O'Meara S, Martyn-St James M. Foam dressings for venous leg ulcers. *Cochrane Database Syst Rev* 2013(5):CD009907. doi: 10.1002/14651858.CD009907.pub2
51. Coleman S, Nelson EA, Vowden P, et al. Development of a generic wound care assessment minimum data set. *J Tissue Viability* 2017;26(4):226-40. doi: 10.1016/j.jtv.2017.09.007
52. Health Service Executive. National best practice and evidence based guidelines for wound managment. Dublin, Ireland: Health Service Executive, 2009.
53. Boaz A, Hanney S, Jones T, et al. Does the engagement of clinicians and organisations in research improve healthcare performance: a three-stage review. *BMJ Open* 2015;5(12):e009415. doi: 10.1136/bmjopen-2015-009415 [published Online First: 2015/12/15]
54. Luker KA, Kenrick M. An exploratory study of the sources of influence on the clinical decisions of community nurses. *J Adv Nurs* 1992;17(4):457-66.
55. McCaughan D, Thompson C, Cullum N, et al. Nurse practitioner and practice nurses' use of research information in clinical decision making: findings from an exploratory study. *Fam Pract* 2005;22(5):490-7. doi: 10.1093/fampra/cmi042
56. O'Leary D F, Mhaolrunaigh SN. Information-seeking behaviour of nurses: where is information sought and what processes are followed? *J Adv Nurs* 2012;68(2):379-90. doi: 10.1111/j.1365-2648.2011.05750.x

For peer review only - http://bmjopen.bmj.com/site/about/guidelines.xhtml

57. Robertson R, Wenzel L, Thompson J, et al. Understanding NHS financial pressures How are they affecting patient care? London: The King's Fund, 2017.
58. Jackson C. Implementing a District and Community Nursing workload tool, to determine safe staffing levels and skill mix in a community care provider organisation: An economic assessment of potential benefits for workforce planning. Kent: Canterbury Christ Church University 2016.
59. Saltman RB. The impact of slow economic growth on health sector reform: a cross-national perspective. *Health Econ Policy Law* 2018:1-24. doi: 10.1017/S1744133117000445
60. Karanikolos M, Mladovsky P, Cylus J, et al. Financial crisis, austerity, and health in Europe. *Lancet* 2013;381(9874):1323-31. doi: 10.1016/S0140-6736(13)60102-6
61. Bussieres AE, Patey AM, Francis JJ, et al. Identifying factors likely to influence compliance with diagnostic imaging guideline recommendations for spine disorders among chiropractors in North America: a focus group study using the Theoretical Domains Framework. *Implement Sci* 2012;7:82. doi: 10.1186/1748-5908-7-82

Funding

This project was funded by the National Institute for Health Research Collaboration for Leadership in Applied Health Research and Care (NIHR CLAHRC) Greater Manchester. The NIHR CLAHRC Greater Manchester is a partnership between providers and commissioners from the NHS, industry and the third sector, as well as clinical and research staff from the University of Manchester. The views expressed in this article are those of the authors and not necessarily those of the NHS, NIHR or the Department of Health.

Contributors

NC, JD and TG conceived the idea and design for the overall project, PW contributed to further development of the study design. TG, PW and JD collected the data. TG and PW were responsible for data analyses. TG created the original draft of the manuscript. All authors contributed to the interpretation of study findings, critical revision of the manuscript for important intellectual content and approval of the final manuscript.

Acknowledgements

The authors are grateful to many colleagues within the NIHR CLAHRC Greater Manchester Wound Care Programme, who have assisted with and supported this work. We thank all participants for giving their time, sharing their views and for their enthusiasm throughout

For peer review only

For peer review only - http://bmjopen.bmj.com/site/about/guidelines.xhtml

Conflicts of interest

None declared

Data sharing statement

Requests for access to data should be addressed to the corresponding author.

For peer review only

For peer review only - http://bmjopen.bmj.com/site/about/guidelines.xhtml

The University of Manchester

National Institute for Health Research

Appendix 1: What Factors Influence Community Wound Care: Healthcare Professional Focus Group Questions

Questions	Prompts for further exploration	TDF Domains
How do you decide which dressing or treatment to use for which patient?	What factors contribute and how is it/are they obtained? Please provide examples to explain answers<br>• Knowledge and skills,<br>○ Under/post grad training/regular updates<br>○ Peers/networking<br>○ Experience/Expertise<br>○ Preferences<br>○ Specialist support<br>○ Conferences/seminars<br>○ Pharmaceutical reps/fact sheets<br>• Research evidence<br>○ Reading journals<br>○ On-line search<br>○ National guidelines<br>○ Communicated via wound care specialists<br>• Patient and carers' influence<br>○ Lifestyle<br>○ Adherence<br>○ Choice/Preference<br>○ Anatomical factors /dressing suitability for foot in mobile patient	Skills Knowledge<br><br>Social professional role and identity<br><br>Behavioral Regulation<br><br>Environmental context and resources<br><br>Beliefs about capabilities<br><br>Memory, attention and decision processes<br><br>Beliefs about consequences<br><br>Motivation and goals<br><br>Emotion
Are there any environmental (organisational or resource based) factors that influence your prescribing practice?	Are the following enablers or constraints?<br>• Processes e.g. having a formulary in place?<br>○ If a formulary is in place is ordering from it mandatory?<br>• Product cost?<br>• Value for money?<br>○ Are some products worth paying more for e.g. silver/soft silicon?<br>○ What additional benefits do they provide?<br>○ How do you justify the additional cost?<br>• Product availability?<br>• Product knowledge? Why choose one product over another?r<br>• Memory (considering the number of products available)?<br>• Training? Competence?<br>• Caseload? Automony?<br>• Team support?	Behavioral Regulation<br><br>Environmental context and resources<br><br>Knowledge<br><br>Skills<br><br>Beliefs about capabilities<br><br>Memory, attention and decision processes<br><br>Beliefs about capabilities

For peer review only

The University of Manchester

National Institute for Health Research

Questions	Prompts for further exploration	TDF Domains
Do you have any influence on what products are included in your Trust formulary?	How?<br><br>Who else is involved?<br><br>If not you – who decides	Social professional role and identity<br><br>Skills<br><br>Environmental context and resources<br><br>Beliefs about capabilities<br><br>Nature of the behaviors
Have other people or situations ever caused you to change your wound care practices?	An incident? What happened?<br><br>Service reconfiguration? Why was this necessary?<br><br>A change in policy? Why was the policy changed? How was the change implemented?<br><br>A colleague? An expert in the field?<br><br>What processes are in place to share practice relating to product usage?<br><br>Do current networks adequately promote shared cared between teams and services? If not what do you think needs to be done to improve this?	Behavioral Regulation<br><br>Environmental context and resources<br><br>Nature of the behaviors<br><br>Social influences
How do you know you are/your service or your trust is making the right decisions regarding product use and service delivery?	Have you completed any audits of clinical care, clinical outcomes or service outcomes?<br>• Are prescribing practices audited? If so how frequently?<br>• Are prescribing skills audited? If so how frequently?<br>• Are prescribing skills regularly monitored/ appraised? If so how frequently?<br>• Do you have a PDP? Do attend regular personal development reviews? How often?<br>• Do you receive regular updates at trust or service level regarding service delivery achievements?<br>What other measures are in place to monitor prescribing practices?	Behavioral Regulation<br><br>Motivation and goals

For peer review only

The University of Manchester

National Institute for Health Research

Now you have had time to look at the wound care product expenditure………..		
**Questions**	**Prompts for further exploration**	**TDF Domains**
Are the overall figures what you were expecting?	Is the overall spend higher or lower than you were expecting?<br><br>What do you think has caused the difference (if any)?<br><br>How do you feel about the differences or similarities with neighboring Trusts and national figures?	Knowledge<br><br>Beliefs about consequences<br><br>Behavioral Regulation<br><br>Environmental context and resources
Does the expenditure for any particular product group surprise you?	Are you surprised that it is low or high expenditure?<br><br>Why does it surprise you?<br><br>What do you think has caused this?<br><br>How do you feel about the differences with neighboring Trusts and national figures?	Knowledge<br><br>Beliefs about consequences<br><br>Environmental context and resources<br><br>Behavioral Regulation
Do you think there is over or under use of any product group?	Which group(s)? Is this over or under used?<br><br>What do you think has caused this?<br><br>Prior to seeing the figures, which product groups did you believe were used the most frequently?<br><br>Prior to seeing the figures, which product groups did you believe were used the least frequently?<br><br>How do you feel about the comparison with neighboring Trusts and national figures?	Knowledge<br><br>Skills<br><br>Beliefs about consequences<br><br>Environmental context and resources

For peer review only

The University of Manchester

National Institute for Health Research

Appendix 2. What Factors Influence Community Wound Care: Procurement Focus Group Questions

Introductory statement to clarify the focus: As you all represent different organisations and have different roles, our questions will aim to establish the procurement processes and practices across four community healthcare organisations (that have partnered with CLAHRC GM to deliver this work) and neighbouring partner CCGs. When we say 'local' we are referring to the 14 services involved. We will use prompts to explore each question further so that we have a clear understanding of each of the services involved.

Questions	Prompts for further exploration	TDF Domains
What procurement processes are in place locally?	Stock list only? Prescribing only? Combination?<br><br>How is information about the systems/practices circulated to community and primary care staff? Are communication channels monitored?<br><br>Do Trusts/CCGs have a formulary?<br>• How are they compiled/updated?<br>• Who is involved?<br>• Who influences what is included?<br><br>What are the drivers for changing processes/practice? Do you have examples?<br>• Incidents?<br>• Audit findings?<br>• Service reconfiguration?<br>• Research evidence?<br>• Lessons learnt from other services?	Environmental context and resources<br><br>Knowledge<br><br>Skills<br><br>Social professional role and identity<br><br>Social influences

For peer review only

MANCHESTER 1824 The University of Manchester

NHS National Institute for Health Research

Questions	Prompts for further exploration	TDF Domains
Are there any factors that influence procurement decisions?	Are the following enablers or constraints?<br>• Product cost?<br>• Product availability?<br>• Product knowledge?<br>• Memory (considering the number of products available)?<br>• Training?<br>• Competence?<br>• Team support?<br>• Company reps<br>○ access monitored/unmonitored<br>○ Incentives	Behavioral Regulation<br><br>Environmental context and resources<br><br>Knowledge<br><br>Skills<br><br>Beliefs about capabilities<br><br>Memory, attention and decision processes
What locally agreed CQUINs/policies are in place for wound care management?	What do these entail?<br><br>Are there any requirements to conduct audits to monitor adherence to agreed wound care management pathway?<br>• Six monthly/yearly?<br>• What are the consequences for poor adherence<br><br>Do they include incentive schemes?<br>Does they include a commitments to reduce spend e.g. reduce silver dressings spend.<br>Do these include a commitment to undertake an ongoing programme of educational training to community or primary care staff on agreed wound management pathway (inc formulary)?<br><br>Do they include a commitment to benchmark with other organisations? (Which?)	Behavioral Regulation<br><br>Environmental context and resources<br><br>Beliefs about consequences<br><br>Motivation and goals<br>Skills
Do healthcare professionals follow policy when ordering /prescribing wound care products?	What percentage of products are ordered/prescribed 'off formulary'<br><br>Are there specific products that are prescribed regularly 'off formulary'?<br><br>Are there any incentives to promote good prescribing/ordering practices?<br><br>If a stock list exists, are stock products sometimes prescribed rather than ordered from the stock list?<br><br>What are the consequences of not following policy/ordering off formulary?<br><br>What do you think can be done to improve adherence to policy?	Behavioral Regulation<br><br>Environmental context and resources<br><br>Beliefs about consequences

For peer review only

The University of Manchester

NHS National Institute for Health Research

Now you have had time to look at the wound care product expenditure………..		
**Questions**	**Prompts for further exploration**	**TDF Domains**
How do you feel about the differences or similarities in expenditure locally compared to the national spend?	Is the overall spend higher or lower than you would like it to be?<br><br>How do you feel about the differences or similarities with the rest of GM and the national figures?<br><br>If overall spend is higher than average what do you think can be done to reduce the spend?	Knowledge<br><br>Beliefs about consequences<br><br>Behavioral Regulation<br><br>Environmental context and resources
Is the expenditure for any particular product higher or lower than you think it should be?	Why do to think expenditure is higher or lower?<br><br>Which type of HCP is contributing to the high/low expenditure?<br><br>How does this compares with the rest of the region and the national figures?	Knowledge<br><br>Beliefs about consequences<br><br>Environmental context and resources<br><br>Behavioral Regulation
Do you think there is over or under use of any product group?	Which group(s)? Is this over or under use?<br><br>What do you think has caused this?<br><br>How does this compare with regional and the national figures?	Knowledge<br><br>Skills<br><br>Beliefs about consequences<br><br>Environmental context and resources

For peer review only

BMJ Open

WHAT FACTORS INFLUENCE COMMUNITY WOUND CARE IN THE UK? A focus group study using the Theoretical Domains Framework

Journal:	*BMJ Open*
Manuscript ID	bmjopen-2018-024859.R1
Article Type:	Research
Date Submitted by the Author:	01-Mar-2019
Complete List of Authors:	Gray, Trish; University of Manchester, Division of Nursing, Midwifery and Social Work, School of Health Sciences, Faculty of Biology, Medicine and Health<br>Wilson, Paul; University of Manchester, Alliance Manchester Business School<br>Dumville, Jo C.; University of Manchester, Division of Nursing, Midwifery and Social Work, School of Health Sciences, Faculty of Biology, Medicine and Health<br>Cullum, Nicky; The University of Manchester, Division of Nursing, Midwifery and Social Work, School of Health Sciences, Faculty of Biology, Medicine and Health
**Primary Subject Heading**:	Qualitative research
Secondary Subject Heading:	Health services research
Keywords:	QUALITATIVE RESEARCH, FOCUS GROUP, WOUND MANAGEMENT, HEALTHCARE PROFESSIONAL, HEALTHCARE QUALITY, THEORETICAL DOMAINS FRAMEWORK

SCHOLARONE™
Manuscripts

WHAT FACTORS INFLUENCE COMMUNITY WOUND CARE IN THE UK? A focus group study using the Theoretical Domains Framework

Corresponding Author

Trish Gray
Division of Nursing, Midwifery and Social Work; School of Health Sciences;
Faculty of Biology, Medicine and Health.
The University of Manchester, Room 6.332, Jean McFarlane Building,
Oxford Road, Manchester, M13 9PL.
Email: Trish.Gray@manchester.ac.uk
Tel: (+44) (0)161 306 7849

Authors:

Trish A Gray,[1,3.] Paul Wilson,[2,3] Jo C Dumville,[1,3] Nicky A Cullum.[1,3,4]

Affiliations

1 Division of Nursing, Midwifery and Social Work, School of Health Sciences, Faculty of Biology, Medicine and Health, University of Manchester, Manchester, UK

2 Alliance Manchester Business School, University of Manchester, Manchester, UK

3 NIHR CLAHRC Greater Manchester 3rd Floor Mayo Building, Salford Royal NHS Foundation Trust, Stott Lane, Salford, Manchester, UK

4 Research and Innovation Division, Central Manchester University Hospitals NHS Foundation Trust, Grafton Street, Manchester, UK

Word Count

Abstract 284
Main text: 4707

KEY WORDS

Qualitative research, focus group, wound management, healthcare professional, healthcare quality, Theoretical Domains Framework.

For peer review only

For peer review only - http://bmjopen.bmj.com/site/about/guidelines.xhtml

ABSTRACT:

Objectives: Research has found unwarranted variation in wound care across community services in the North of England, with underuse of evidence-based practice and overuse of interventions where there is little or no known patient benefit. This study explored the factors that influence community wound care to develop a deeper understanding of the current context of wound care and variation in practice.

Design: Qualitative focus group study using The Theoretical Domains Framework to structure the questions, prompts and analyses.

Setting: Community healthcare settings in the North of England, UK.

Participants: Forty-six clinical professionals who cared for patients with complex wounds and eight non-clinical professionals who were responsible for procuring wound care products participated across six focus group interviews.

Results: We found the TDF domains: Environmental context and resources, Knowledge, Skills, Social influences and Behaviour regulation to best explain the variation in wound care and the underuse of research evidence. Factors such as financial pressures were perceived as having a negative effect on the continuity of care, the availability of wound care services and workloads. We found practice to be mainly based on experiential knowledge and personal preference and highly influenced by colleagues, patients and the pharmaceutical industry though not by research evidence.

Conclusions: Our study provides new insight into the role that experiential learning and social influences play in determining wound care and on the limited influence of research. Workforce pressures and limited resources are perceived to impede care by reducing patient access to services and the ability to provide holistic care. Participative collaboration between university and healthcare organisations may offer a supportive route to addressing issues, implementing sustainable changes to practice and service delivery and a resolute commitment to research use amongst clinical professionals.

For peer review only

For peer review only - http://bmjopen.bmj.com/site/about/guidelines.xhtml

Article Summary

Strengths and Limitations of this study

- This focus group study is the first to explore the factors that influence wound care and the reasons for known variation in practice.
- Employing a qualitative methodology provided new insight into the role experiential learning and social influences play in determining clinical and procurement choices.
- The focus group design stimulated discussion allowing participants to examine their own and others' views and experiences.
- The Theoretical Domains Framework provided a theoretical structure for developing a deeper understanding of wound care delivery.
- The sample was taken from community healthcare organisations in the North of England, inclusion of participants from a larger geographical population may have provided different views.

MAIN TEXT

Introduction

People with complex wounds (open wounds, such as foot, leg and pressure ulcers, burns, open trauma and surgical wounds that are difficult to heal),[1 2] are more likely to be elderly and living with multimorbidity.[3] In the UK, the management of people with complex wounds[1 2] is mainly carried out in patients' homes or community clinics by community nurses with advice and support from specialist teams (nurses and medics with expertise in tissue viability, burns, vascular medicine or dermatology). Podiatrists also play a vital role in managing foot wounds, often working in conjunction with community nurses.

Wound care normally begins with a comprehensive assessment of the person and their wound before implementation of appropriate interventions.[4] Specific wound-related assessments include ankle brachial

For peer review only - http://bmjopen.bmj.com/site/about/guidelines.xhtml

pressure index (ABPI) for people with venous leg ulcers. Wound treatment may involve wound cleansing followed by dressing to manage exudate and protect the wound. Whilst dressings are used widely across wound types, with many different options available for use, there is currently no evidence that one dressing type is more clinically or cost effective than another, even in the case of relatively expensive anti-microbial dressings. In contrast there are effective first line treatments which should be widely used, such as the use of compression therapy for venous leg ulceration which is known to reduce time to wound healing [5 6]

As part of a wider programme of wound care research funded by the National Institute for Health Research Collaboration for Leadership in Applied Health Research and Care (NIHR CLAHRC) Greater Manchester, we conducted a survey to assess how healthcare professionals managed wound care across five community healthcare organisations in the North of England.[7] The findings are discussed in more detail elsewhere[7] but in summary the survey revealed unwarranted variation in clinical practice, with general underuse of Doppler-aided measurement of ABPI,[8] underuse of compression therapy and [9]). potential overuse of antimicrobial dressings.[6] In the UK, variations in wound care are being recognised and addressed with initiatives such the Leading Change, Adding Value Nursing and Midwifery Framework[10 11] however, there has been little formal exploration of drivers for this variation in the delivery of wound care and barriers to implementing the findings from current research evidence. In turn there is little intelligence to guide further research implementation and bring about meaningful practice change with the aim of maximising patient benefit.

Our aim was to identify and explore factors that influence the current delivery of wound care in community settings, where delivery is used to describe wound assessment and treatment. We wanted to better understand the current context of community wound care and how research evidence informs care delivery. We were keen to explore clinical viewpoints and also, because of possible factors linked to the availability and use of wound products with a limited evidence-base, we involved non-clinical staff responsible for procurement processes.

For peer review only

For peer review only - http://bmjopen.bmj.com/site/about/guidelines.xhtml

Methods

Design

We conducted six focus group interviews to explore the factors that influence the delivery of wound care. The Theoretical Domains Framework (TDF) was used to structure the questions, prompts and analyses.[12 13] The TDF provides a theoretical lens through which to view cognitive, affective, social and environmental factors that could potentially influence behaviour.[14] It has been used extensively across a range of clinical areas.[15-17] Its constructs are grouped into 14 discrete domains.[12] The TDF is presented in Table 1 showing the domains, definitions and examples of behaviours related to wound care and wound product procurement.

For peer review only - http://bmjopen.bmj.com/site/about/guidelines.xhtml

Table 1: The Theoretical Domains Framework: domains, definitions and examples of behaviours related to wound care and wound product procurement

Domain	Definition	Examples of wound care and wound product procurement behaviors
Knowledge	An awareness of the existence of something.	Knowledge of wound types, wound aetiology, risk factors, wound product types and influenced by education, experience, research
Skills	An ability or proficiency acquired through practice.	Ability to complete a comprehensive wound assessment, specific assessments such as ABPI, apply compression bandages/stockings, managing procurement processes effectively
Social/Professional role and identity	A coherent set of behaviors and displayed personal qualities of an individual in a social or work setting.	Carrying out a clinical or procurement role according to job description, communicating and working appropriately and effectively with other clinical or non-clinical professionals
Beliefs about capabilities	Acceptance of the truth, reality, or validity about an ability, talent, or facility that a person can put to constructive use.	Being confident in making the right decisions about care for patients with complex wounds, confidence in negotiating skills for product procurement
Optimism	The confidence that things will happen for the best or that desired goals will be attained.	Confidence that care provided will achieve cure/manage wounds effectively, confident that most cost effective products can be purchased
Beliefs about consequences	Acceptance of the truth, reality, or validity about outcomes of a behavior in a given situation.	Having realistic views about patient adherence to treatment plans and healing rates of complex wounds
Reinforcement	Increasing the probability of a response by arranging a dependent relationship, or contingency, between the response and a given stimulus.	Support of colleagues, team work, wound care provided has produced the desired goal, research evidence that interventions work
Intentions	A conscious decision to perform a behaviour or a resolve to act in a certain way.	To practice according to a care plan, national/international guidelines
Goals	Mental representations of outcomes or end states that an individual wants to achieve.	Setting goals for wound healing, improving patient adherence, achieving competence for a new skill
Memory, attention and decision processes	The ability to retain information, focus selectively on aspects of the environment and choose between two or more alternatives.	Ability to remember wound care information learnt, dressing specifications considering the wide choice, making decisions based on evidence
Environmental context and resources	Any circumstance of a person's situation or environment that discourages or encourages the development of skills and abilities, independence, social competence and adaptive behavior.	Organisational structures, procedures and processes, workload pressures, staff shortages, funding constraints, service cuts, procurement processes, product cost, product availability,
Social influences	Those interpersonal processes that can cause individuals to change their thoughts, feelings, or behaviors.	Decisions influences by personal, colleagues' patients, pharmaceutical industry preferences, team work and shared care, understanding patients' needs, negotiating product cost
Emotion	A complex reaction pattern, involving experiential, behavioral, and psychological elements, by which individual attempts to deal with a personally significant matter or event.	Coping with wounds that do not heal, managing challenging wounds, dealing with emotions when patients with complex needs deteriorate or die
Behavioral regulation	Anything aimed at managing or changing objectively observed or measured actions.	Formulary to guide (restrict) prescribing and procurement choices, audits of practice and procedures

122 Adapted from © Cane et al.; licensee BioMed Central Ltd.

For peer review only - http://bmjopen.bmj.com/site/about/guidelines.xhtml

Participants and settings

Purposive sampling was used to ensure that we recruited participants with relevant clinical and/or procurement experience. Eligibility included community-based clinical professionals who cared for patients with complex wounds or non-clinical professionals who were involved in the procurement of wound care products. Clinical professionals included community nurses, podiatrists, tissue viability or burns specialist nurses, wound research nurses and clinical nurse managers (who had a clinical role, managed a team of community nurses and were responsible for wound product procurement decisions). Non-clinical professionals included: medicines optimisation pharmacists, procurement leads, procurement advisors and medicines management leads.. There were five multidisciplinary focus group interviews for clinical professionals; one for each participating provider organisation. Four were drawn from provider organisations in one defined geographical area with a fifth conducted in a different geographical area but similar urban conurbation in the North of England; chosen for its well-established links with university researchers as a comparison to the other organisations where collaborative partnerships with university researchers were in their infancy. A separate focus group interview was held for non-clinical professionals. As the themes for clinical and non-clinical focus group interviews differed, we chose this format to maintain focus and create an optimum environment for free flowing discussions. g Potential participants were identified through contacts developed as part of the NIHR CLAHRC GM wound care programme and were approached via email, telephone or face-to-face meeting. Focus group interviews were held locally to participants' work place in a healthcare setting or conference centre.

As participants were drawn from a relatively homogeneous population and the interview schedules were focused on specific aspects of wound care and wound product procurement, we anticipated that we would reach data saturation within three to four focus group interviews, however, we aimed to recruit 50-60 participants in total across the six groups (8 to 10 participants per group), based on recommendations from existing literature[18-21] to incorporate all partner provider organisations using the format described above.

For peer review only - http://bmjopen.bmj.com/site/about/guidelines.xhtml

Data collection

The format was similar for all focus group interviews; they were facilitated by a lead (TG) with one or two co-facilitators (PW and JD). All facilitators were experienced researchers and familiar with the evidence base for wound care. A fourth member of the research team took field notes. Before the session began, participants were asked to complete a brief demographic questionnaire to clarify their academic and professional qualifications and wound care/product procurement experience as relevant; these data were used to describe the participants involved and were not linked to particular responses or quotes. Each session was audio recorded with recordings deleted following verification of anonymised transcripts.

Procedure

The discussion explored specific behaviours linked to the TDF domains and reactions to site-specific, regional and national procurement data using the questions and prompts outlined in Appendix 1. Clinical professionals were encouraged to think about factors that from their experience, enable or hinder the delivery of wound care, relating their answers to their own experiences. Through prompts we probed further, allowing participants' reactions to unfold, giving them the opportunity to explore their own and others' views. We continued to prompt if responses were not spontaneously offered to encourage full participant engagement. The focus group interview for non-clinical professionals followed an identical format with the questions more related to procurement systems and procedures

(Appendix 2). Interview schedules were piloted by specialist nurses, clinical managers and a procurement lead, after which minor amendments were made. Respondents validated the accuracy and completeness of the findings[22] following a verbal summary (taken from the field notes) at the end of each focus group interview and a post-analysis report sent via email.

For peer review only - http://bmjopen.bmj.com/site/about/guidelines.xhtml

Ethical considerations

Ethics approval was sought and granted from the University of Manchester Research Ethics Committee (Refs 15272, 15327 and 2017-0559-1767) and HRA approval was sought and granted (Refs IRAS 174691, 184865 and 219918). Written informed consent was obtained from all participants.

Patient and Public Involvement

Patients and the pubic were not involved in the interviews, however, views expressed by members of the NIHR CLAHRC Greater Manchester Wounds Research PPI Forum regarding their experiences with healthcare professionals and wound care services were used to inform some of the questions and prompts for the focus group discussions.

Data Analysis

Quantitative data were stored in SPSS (IBM version 22). Demographic variables are expressed in frequencies, means and standard deviations where distributions are normal, and medians and range when skewed. Qualitative analysis followed a seven-step process in line with the framework method (Figure 1).[23-25]

Findings

Participant characteristics

Sixty participants were invited to attend one of six focus group interviews (mean duration: 106 minutes). Fifty-four participants attended whilst nine invited participants could not attend due to other clinical commitments or annual leave (three of whom nominated colleagues to attend in their place). Participants comprised 46 clinical professionals (ten specialist nurses (19%), 25 community nurses (46%), seven podiatrists (13%), three clinical managers (5%) and one research nurse (2%)) and eight non-clinical professionals (15%). Wound care experience was extensive (mean 14.6 years, SD 8.8) amongst clinical professionals (Table 2).

For peer review only - http://bmjopen.bmj.com/site/about/guidelines.xhtml

Table 2. Participant Characteristics (n=54)	N (%)
Gender	
Male	7 (13)
Female	47 (87)
Role group Clinical professional	
Specialist nurse	10 (19)
Community nurse	25 (46)
Research nurse	1 (2)
Clinical Manager	3 (5)
Podiatrist	7 (13)
**Role group *Non-clinical professional***	8 (15)
Highest Academic Qualification	
MSc	6 (11)
BSc/BA (Hons)	27 (50)
PG Diploma	11 (20)
PG Certificate	2 (4)
Vocational Qualification	5 (9)
A level	3 (6)
Years in current role	**Mean (SD)**
Clinical professional	8.6 (7.4)
Non-clinical professional	4.7 (4.3)
Years of wounds care/procurement experience	
Clinical professional	14.5 (8.8)
Non-clinical professional	5.7 (6.4)
Attended a wound care update in last 12 months (n=46)	***N (%)***
Yes	15 (33)
No	31 (67)
Attended wound procurement update in last 12 months (n=8)	
Yes	1 (13)
No	7 (88)

Key themes identified within relevant domains

Five TDF domains dominated: *Environmental context and resources*, *Knowledge, Skills, Social influences* and *Behaviour regulation*. The domains of knowledge and skills were closely linked and frequently overlapped, therefore, we combined these. We did not code any source data to the domains of *Emotion* and *Intentions* and found the remaining six domains to overlap with the five dominant domains. We have therefore, focused on the five key domains which best explain the variation in wound care and the underuse of research evidence. The coding tree (Figure 2) demonstrates the relationships between domains and sub-themes.

For peer review only

Environmental context and resources

Delivery of care

Clinical professionals across all groups expressed feeling the pressure of increased workloads. Some participants said they were working more intensely and without breaks, constantly feeling anxious that they may have missed something as time was limited between patient consultations. They reported that there was an increase in sick leave, experienced colleagues were leaving and their roles were left vacant.

"You haven't got the same skill base any more. We haven't got the same expertise, we're losing our experienced link nurse this week, and we haven't really got anybody with that level of skill in wounds to take her place........we've got 30 vacancies at the moment that haven't been filled" (Clinical manager)

Community nurses reported that specialist clinics were being cut, and patients previously seen in dedicated leg ulcer clinics by nurses with specialist knowledge, were now visited at home by what participants suggested to be understaffed community nursing teams.

"Physically running the clinic was based on when there was about six or seven [leg ulcer specialist] staff ... when it was a leg ulcer service. There's only two of us so we haven't got the capacity to cover those let alone do all the home visits." (Community nurse)

Community nurses and podiatrists voiced concern that undue time was spent gathering required patient information due to poor referral information supplied by hospital staff.

"You constantly are ringing because they'll [ward staff] *put* [on the referral] *"care of wound", but ...... what wound have they got? What operation have they had? What would you like me to do with it? It's very, very poor."* (Community nurse)

Variation in care and services

Many clinical participants attributed variation in the patterns of care delivery to realignment of services due to reduced funds. The majority of clinical professionals reported that specialist leg ulcer clinics had been cut resulting in a greater number of home visits for community nurses. One specialist nurse however, reported that leg ulcer care within her service had recently been taken from the community nurses' workload and assigned to a specialist team (this was the only community service within the organisation to realign services in this way). Participants from another organisation, reported that practice nurses (nurses based in a general practitioner

For peer review only - http://bmjopen.bmj.com/site/about/guidelines.xhtml

practice providing primary care for a local population) managed mobile patients with wounds, whilst community nurses cared for housebound patients with more complex health needs. This changed model of service delivery was felt by community nurses to have eased their workload.

Participants from organisations that managed both hospital (acute) and community services felt that resourcing prioritised the acute service at the expense of the community service. Participants made reference to the differences between resources available in acute care that were limited or unavailable in the community; this included wound care products and digital technology.

"I just don't feel the acute side has got a grip at all on community services in terms of what we do…I mean, I do a specialist [acute] clinic on a Tuesday morning and have access to all sorts of dressings. And I come back into the community….and we're very limited, we've got one foam [dressing] *that we can use."* (Podiatrist)

Clinical participants viewed access to photographic equipment as a valuable resource that allowed images of wounds to be sent to a podiatrist or specialist nurse for rapid diagnosis and care planning, however, only healthcare professionals with access to hospital photographic equipment could make use of this service. One specialist nurse apologised for using photographic equipment that the community nurses within her organisation did not have access to.

"I do [take photographs of wounds]. I've got a camera. Sorry. It is downloaded onto a programme at the hospital. So that's probably why [I have access to it]." (Specialist nurse)

Variation in product procurement

Participants reported a variety of wound care product procurement processes; some (across two provider organisations) obtained all products via prescription, others (across two provider organisations) used a combination of prescribing and stock purchase and one group (one organisation) operated a total stock purchase system. All participants noted the local use of wound care formularies (a locally developed list of recommended products), to guide prescribing or purchasing decisions however through discussion it was recognised that the products listed and the number of product available varied across formularies. One

For peer review only - http://bmjopen.bmj.com/site/about/guidelines.xhtml

organisation had a very restrictive formulary and monitored use closely; participants found this restrictive formulary enabled them to choose appropriate products.

"I think it's an enabler, … there are so many [dressings to choose from] *you can go completely for something that costs so much and something that wouldn't be right … but having that formulary means that we know what we can choose."* (Community nurse*)*

Knowledge and skills

Education and training

All nurse participants agreed that there was a limited amount of wound care education for student nurses and that most wound care knowledge and skills were gained through community placements rather than in the classroom. Only specialist nurses had attended a university-based post-registration wound care course. In contrast, podiatrists received regular undergraduate and postgraduate wound care education. . All clinical professionals viewed wound care knowledge across other services (hospital, primary care and nursing homes) to be poor, which increased their workload if aspects of care, documentation or prescription information were incomplete.

Specialist nurses reported that due to workforce pressures, in-house courses they offered were often cancelled or attendance was poor. Due to these difficulties, specialist nurses relied on the pharmaceutical industry to provide wound product training sessions.. Concerns were raised particularly by the non-clinical professionals that the educational contributions of industry representatives were highly likely to favour their own products.

"But then at the same time they'd spy the competition and they'd basically suggest that their products are equivalent to those products that were already on the shelf …….and then we were inundated with requests for new products.*"* (Non-clinical professional)

Use of research evidence

Only participants from the provider organisation with a history of collaborative wound care research indicated that they actively sought to keep up to date with research. Specialist nurses from this focus group talked about their established links with university researchers and their involvement in co-producing wounds research with academics. They discussed disseminating relevant research findings through electronic newsletters, workshops

For peer review only - http://bmjopen.bmj.com/site/about/guidelines.xhtml

and meetings with community staff and where capacity allowed, staff were supported to implement research findings. Participants reported that their organisation was highly research active in wound care; clinical professionals had participated in research that found compression stockings to be more cost-effective than compression bandages for people with venous leg ulcers[9] and they subsequently implemented the findings into practice. The remaining participants viewed research with caution, they found very little time to search for evidence or be involved in research.[6 26 27]

"I can't know everything about all dressings, and therefore you often stick to what you know and you don't often have time to look at research." (Non-clinical professional)

"And as healthcare professionals it's not built into our contracts to do research…there's no time put aside." (Specialist nurse)

Social influences

Team work

The importance of good teamwork was frequently emphasised and acknowledged by all participants. Much of the sharing of experiences was conducted informally. Clinical professionals reported that team support alleviated some of the current workload pressures and shared care was viewed as a valuable method for joint decision making. Participants from one focus group only reported the existence of wound care link nurses whose role was to cascade new information, new research evidence and product updates from specialist nurses to their colleagues. However, capacity issues were affecting the scope of this role.

Industry and patient influence

As referred to above, all participants were concerned about the influence of the pharmaceutical industry it was felt that this influence varied depending on the capacity of specialist nurses to limit access and monitor training sessions. Non-clinical participants were particularly and negatively vociferous about the influence of pharmaceutical representatives yet viewed the role of policing any promotional activity as a specialist nurse responsibility.

For peer review only

For peer review only - http://bmjopen.bmj.com/site/about/guidelines.xhtml

"You can police this a little bit more in acute, can't you, but in the community we were fighting a losing battle with the reps when they're just given free range to provide training."(Non-clinical professional)

Participants were very aware of the influence that patients have on wound care, which at times caused difficulty finding a suitable dressing that met patients' expectations. Participants reported that some patients removed dressings earlier than necessary if minor staining appeared. Participants were mindful that careful assessment and monitoring patients' adherence to therapy was necessary when making product choices. Participants also found that patients searched for information on the internet in an attempt to influence product decisions.

"She [the patient] *read that honey was good and she thought I'll go and buy my own…. and swore it did the trick, so who are we to argue with her?"* (Community nurse)

Behaviour regulation

Monitoring and inappropriate prescribing Community nurses reported that antimicrobial dressings (particularly silver-impregnated dressings) were used for individual patients for a two-week trial period and then reviewed, however, they acknowledged that if use was not closely monitored there was potential for misuse. Non-clinical professionals and specialist nurses were aware of the high expenditure on antimicrobial dressings but acknowledged difficulties in monitoring effectively and providing adequate training and support due to capacity issues.

"Silver spend is still a problem and it's a long-term……I think there's still habitual use, district nurses having the time to stop and think and review and stop a treatment rather than continue." (Specialist nurse)

Specialist nurses reported that general practitioners regularly prescribed high cost antimicrobial dressings for nursing home residents. The prescription for these dressings would often be repeated without review unless the resident was referred to a specialist nurse. There was an opinion amongst participants that some prescribing of silver dressings may be accidental because dressings are listed alphabetically in some prescribing platforms and silver dressings appear first (as they are denoted by the chemical symbol for silver, 'Ag').

For peer review only - http://bmjopen.bmj.com/site/about/guidelines.xhtml

Discussion

We believe this is the first study to explore factors influencing community wound care whilst seeking to understand the reasons for known variation in practice. Overall, participants described a challenging working environment, with influences such as workforce shortages and diminishing treatment resources having a marked effect on continuity of care, patient access to services and workload. Clinical practice seemed to be predominantly based on experiential knowledge, personnel preference and to be highly influenced by colleagues, patients and the pharmaceutical industry.

Workforce pressures and diminishing resources

Wound care services were described by participants as a working environment characterised by increasing time pressures and diminishing resources. Clinic sessions had been cut, resulting in an increase of home visits for community nurses to non-housebound patients. Roles were perceived as becoming task orientated which was felt to dilute the quality of care. Participants reported there was a rise in sickness, colleagues were leaving for less pressured roles and vacancies were not being filled. The UK has fewer nurses relative to the population than many EU countries.[28] The number of community nurses is falling, with an estimated vacancy rate of 9.4%.[29] Forty percent of experienced nurse positions (Band 6 and above) are vacant,[30] therefore, the majority of staff currently providing physical and emotional care for older people are low paid and have few qualifications.[31] Community nurses are having to work longer hours and more intensely to protect patient care which is leading to high levels of stress, low morale and increasing absence due to sickness.[32-34] Championing flexible career pathways with clear progression from entry to doctoral studies and beyond[35] and valuing the role of the healthcare assistant by identifying opportunities for learning and development[36] may improve staff recruitment. The development of integrated teams and the introduction of combined hospital and community posts to standardise practice, improve care coordination and vary work experiences could have a positive effect on retention rates.[30 37 38] Improved information technology allowing more effective time management and

For peer review only - http://bmjopen.bmj.com/site/about/guidelines.xhtml

facilitating shared care between services, including remote consultations, may increase capacity for specialist teams allowing a greater number of patients to receive input by highly skilled healthcare professionals.[39 40]

Experiential learning and social influences

All nursing participants agreed that there was a lack of wound care education in basic nurse education. Wound care skills were learnt during community but not hospital placements. This was verified by participants' reference to insufficient information from hospital nursing and medical staff on referral forms and via telephone calls which cause delays in assessment and frustration for community nurses. Whilst all specialist nurse teams offered on-going wound care training to community nurses, cancellation or poor attendance frequently occurred due to staff shortages. By contrast, podiatrists' viewed their wound care education to be strong before and after qualification.

In the light of the current workforce issues and the difficulties community nurses had updating their wound care knowledge, other strong influences played a significant role in wound care choices such as personal, colleague and patient preferences as well as the influence of pharmaceutical company representatives. This influence can drive variation in dressing and treatment choice depending on the amount of access pharmaceutical representatives have to healthcare settings and clinical professionals' attitude to the information they provide.[41] The ongoing cuts to continued professional development funding in the UK since 2015 may lead to greater dependence on the pharmaceutical industry for training and 'education' which is problematic due to companies' vested interests in the use of specific products.[42 43] Inter-professional education may break down professional boundaries and provide opportunities for mutual learning and joint solutions across professional groups and specialties.[44] Further investment into evaluated training interventions that are of high quality and independent is warranted to ensure education is consistent and effective; providing healthcare professionals with the confidence to make the right decisions to improve continuity and quality of care.[36 45-47]

For peer review only - http://bmjopen.bmj.com/site/about/guidelines.xhtml

The influence of research on wound care

Using research to guide product choice

There is a plethora of wound products available for use but, as several Cochrane systematic reviews have shown, there is a paucity of research evidence showing that products are clinically effective.[6 48-53] Despite this, product use and expenditure grow; particularly antimicrobial dressing use, where no compelling evidence or guideline recommendations exist to support routine use (Hussey et al., 2018 manuscript submitted for publication).

We found that a restrictive formulary was viewed as enabling better patient management, particularly if guidelines accompanied the formulary. Community nurses found a formulary and guidance gave them more assurance that they were making the right decisions and specialist nurses found formularies reduced inappropriate product choices and assisted in standardising product use across their service. For the majority of organisations, however, the formulary acted as guidance only and 'off-formulary' prescribing could occur without restriction unless resources were available to monitor prescribing behaviour closely. National guidelines exist to guide the use of specific products,[4 6 26 27] however, national standards to guide choice across the range of wound care products would reduce variation of product use and guide more rational prescribing.[54]

Engagement in research

Research was raised as a factor influencing wound care in only one, highly research-active provider organisation. In this site, well-established links with university researchers had been highly influential. Current evidence suggests that there is an association between the engagement of individuals and healthcare organisations in research and improvements in healthcare performance.[55] In the other sites, where collaborative links with university researchers were more newly established, research informed decision making was more limited and research generally was viewed with caution. Much of the discussion around acquiring knowledge and skills to inform wound care decisions was related to experiential influences; day-to-day wound

For peer review only - http://bmjopen.bmj.com/site/about/guidelines.xhtml

care experience, watching others and consulting with more experienced colleagues and specialists. This finding is in line with other research showing that experiential learning and the social influence of peers rather than research knowledge are major influencers on nursing practices.[56-58]

If evidence obtained from research is to inform management and practice, robust, long-term strategies to support and facilitate its use will be required. In England, the NIHR funded research that incentivises co-production of research e.g., NIHR CLAHRCs represent an on-going nationwide experiment to close the distance between research production and research use.

Limitations

The main limitation is the sample which was taken from community healthcare provider organisations in the North of England and included only one research-active organisation. Inclusion of participants from a larger geographical population may have provided different views, however, we captured many of the issues affecting healthcare (such as work pressures, staff shortages and limited resources) across the UK[33 59 60] and further afield[61 62] due to the financial healthcare crisis worldwide. We would have preferred to include more than one research-active organisation but due to the limited number of research-active organisations within our geographical area as well as funding and time limitations we could not recruit more. We were able to recruit the recommended number of participants for each focus group but work pressures dictated the range of clinical professionals and for one group there were no podiatrists which may have reduced the diversity of views, for that particular interview. However there was good representation from podiatrists across the other groups ranging from senior management to junior positions. Podiatrists' utterances were coded as equally as other professional groups, therefore, we feel that podiatrists' views have been incorporated adequately. Only one research nurse was recruited but as the research-active organisation was the only organisation to employ wound research nurses it is not surprising that we could only recruit one.[33 59-62]

For peer review only

For peer review only - http://bmjopen.bmj.com/site/about/guidelines.xhtml

A challenge of using the TDF was the overlap across domains such as K*nowledge* and *Skills*, *Beliefs about consequences* and *Social/professional role and identity.* Other authors have reported similar challenges.[17 63] We found coding to be heavily orientated around the *Environmental context and resources* and it took several iterations and meetings between coders (TG and PW) to ensure that our coding had not overlooked other domains. This process was assisted by the coders being experienced researchers from different professional backgrounds with one (PW) having an in-depth knowledge of the TDF. The recently published guide to using the TDF, addresses these and other challenges to promote the use of the TDF to a wider audience.[14]

Finally, our aim in this study has been to surface factors that could potentially explain variations in the delivery of wound care. We of course recognise that wound care is complex and multifaceted involving a wide range of behaviours. Given this, we recognise that any formal attempts to develop strategies to modify existing practices and behaviours will require a level of granularity beyond what is available in the data presented. Our study does shed light on those domains where those future efforts should focus.

Conclusions

Our study provides new insight into the role experiential learning and social influences play in determining management and treatment choices and on the limited influence of evidence obtained from research. Workforce pressures and limited resources are perceived to impede care by reducing patient access to services, the ability to provide holistic care. Co-production of research evidence through participative collaboration between university and healthcare provider organisations may offer a supportive route to addressing issues, implementing sustainable changes to practice and service delivery and a resolute commitment to research use amongst clinical professionals. .

Figure Legends

Figure 1. Qualitative analysis using a seven step framework method

Figure 2. Coding Tree showing the four salient domains with connected sub themes

For peer review only - http://bmjopen.bmj.com/site/about/guidelines.xhtml

References

1. Cullum N, Buckley H, Dumville J, et al. Wounds Research for Patient Benefit: a 5 year programme of research. *Programme Grants Appl Res* 2016;4(13)
2. Vowden K. Complex wound or complex patient? Strategies for treatment. *Br J Community Nurs* 2005;Suppl:S6, S8, S10 passim. [published Online First: 2005/06/10]
3. Gray TA, Dumville JC, Christie J, et al. Rapid research and implementation priority setting for wound care uncertainties. *PLoS One* 2017;12(12):e0188958. doi: 10.1371/journal.pone.0188958 [published Online First: 2017/12/06]
4. Coleman S, Nelson EA, Vowden P, et al. Development of a generic wound care assessment minimum data set. *J Tissue Viability* 2017;26(4):226-40. doi: 10.1016/j.jtv.2017.09.007
5. Ashby RL, Gabe R, Ali S, et al. VenUS IV (Venous leg Ulcer Study IV) - compression hosiery compared with compression bandaging in the treatment of venous leg ulcers: a randomised controlled trial, mixed-treatment comparison and decision-analytic model. *Health Technol Assess* 2014;18(57):1-293, v-vi. doi: 10.3310/hta18570
6. NICE. Chronic wounds: advanced wound dressings and antimicrobial dressings: Evidence summary [ESMPB2]. London: National Institute for Health and Care Excellence, 2016.
7. Gray TA, Rhodes S, Atkinson RA, et al. Opportunities for better value wound care: a multiservice, cross-sectional survey of complex wounds and their care in a UK community population. *BMJ Open* 2018;8(3):e019440. doi: 10.1136/bmjopen-2017-019440 [published Online First: 2018/03/25]
8. SIGN. Management of chronic venous leg ulcers: A national clinical quideline (120): Scottish Intercollegiate Guidelines Network, 2010.
9. Ashby RL, Gabe R, Ali S, et al. Clinical and cost-effectiveness of compression hosiery versus compression bandages in treatment of venous leg ulcers (Venous leg Ulcer Study IV, VenUS IV): a randomised controlled trial. *Lancet* 2014;383(9920):871-9. doi: 10.1016/S0140-6736(13)62368-5
10. Adderley U, Evans K, Coleman S. Reducing unwarranted variation in chronic wound care. *Wounds UK* 2017;13(4):22-27.
11. England N. Leading Change, Adding Value: A framework for nursing, Midwifery and care staff London, UK: NHS England; 2016 [Available from: https://www.england.nhs.uk/wp-content/uploads/2016/05/nursing-framework.pdf.
12. Cane J, O'Connor D, Michie S. Validation of the theoretical domains framework for use in behaviour change and implementation research. *Implement Sci* 2012;7:37. doi: 10.1186/1748-5908-7-37 [published Online First: 2012/04/26]
13. Michie S, Johnston M, Abraham C, et al. Making psychological theory useful for implementing evidence based practice: a consensus approach. *Qual Saf Health Care* 2005;14(1):26-33. doi: 10.1136/qshc.2004.011155 [published Online First: 2005/02/05]
14. Atkins L, Francis J, Islam R, et al. A guide to using the Theoretical Domains Framework of behaviour change to investigate implementation problems. *Implement Sci* 2017;12(1):77. doi: 10.1186/s13012-017-0605-9
15. Craig LE, Taylor N, Grimley R, et al. Development of a theory-informed implementation intervention to improve the triage, treatment and transfer of stroke patients in emergency departments using the Theoretical Domains Framework (TDF): the T(3) Trial. *Implement Sci* 2017;12(1):88. doi: 10.1186/s13012-017-0616-6
16. Murphy K, O'Connor DA, Browning CJ, et al. Understanding diagnosis and management of dementia and guideline implementation in general practice: a qualitative study using the theoretical domains framework. *Implement Sci* 2014;9:31. doi: 10.1186/1748-5908-9-31
17. Phillips CJ, Marshall AP, Chaves NJ, et al. Experiences of using the Theoretical Domains Framework across diverse clinical environments: a qualitative study. *Journal of multidisciplinary healthcare* 2015;8:139-46. doi: 10.2147/JMDH.S78458 [published Online First: 2015/04/04]
18. Carlsen B, Glenton C. What about N? A methodological study of sample-size reporting in focus group studies. *BMC Med Res Methodol* 2011;11:26. doi: 10.1186/1471-2288-11-26 [published Online First: 2011/03/15]
19. Guest G, Namey E, McKenna K. How Many Focus Groups Are Enough? Building an Evidence Base for Nonprobability Sample Sizes. *SAGE journals* 2016;29(1):3-22. doi: https://doi.org/10.1177/1525822X16639015
20. Krueger RA, Casey MA. Chapter 4. Participants in a Focus Group. Focus Groups: A Practical Guide for Applied Research. 5 ed. Thousand Oaks, C.A.: Sage Publications Inc 2015:79-101.
21. Tang KC, Davis A. Critical factors in the determination of focus group size. *Fam Pract* 1995;12(4):474-5. [published Online First: 1995/12/01]
22. Noble H, Smith J. Issues of validity and reliability in qualitative research. *Evid Based Nurs* 2015;18(2):34-5. doi: 10.1136/eb-2015-102054 [published Online First: 2015/02/06]
23. Bloor M, Frankland J, Thomas M, et al. Focus Groups in Social Research. London: Sage 2001.

For peer review only

For peer review only - http://bmjopen.bmj.com/site/about/guidelines.xhtml

24. Gale NK, Heath G, Cameron E, et al. Using the framework method for the analysis of qualitative data in multi-disciplinary health research. *BMC Med Res Methodol* 2013;13:117. doi: 10.1186/1471-2288-13-117
25. Ritchie J, Spencer L. Qualitative data analysis for applied policy research. In: Bryman B, Burgess R, eds. Analyzing qualitative data. London and New York: Routledge 1994:173-94.
26. NICE. Pressure ulcers: prevention and management Clinical guideline [CG179]: National Institute for Health and Care Excellence, 2014.
27. NICE. Diabetic foot problems: prevention and management [NG19]: National Institute for Health and Care Excellence, 2015.
28. Buchan J, Charlesworth A, Gershlick B, et al. Rising pressure: the NHS workforce challenge: Workforce profile and trends of the NHS in England. London: The Health Foundation 2017.
29. OECD. The Nursing Workforce Health Statistics: Organisation for Economic Co-operation and Development 2017.
30. NHS England. Framework for commissioning community nursing. London: NHS England, 2015.
31. Cornwell J. The care of frail older people with complex needs: time for a revolution: The Sir Roger Bannister Health Sunnit, Leeds Castle. London: The King's Fund, 2012.
32. Ball J, Philippou J, Pike G, et al. Survey of district and community nurses in 2013: report to the Royal College of Nursing. London: Royal College of Nursing, 2014.
33. Maybin J, Charles A, Honeyman m. Understanding quality in district nursing services: Learning from patients, carers and staff. London: The King's Fund, 2016.
34. NQB. Safe, sustainable and productive staffing; An improvement resource for the district nursing service 1ed. London: National Quality Board 2017.
35. Public Health England. Facing the Facts, Shaping the Future: A draft health and care workforce strategy for England to 2027. London: Public Health England, 2017.
36. HEE. Raising the Bar. Shape of Caring: A Review of the Future Education and Training of Registered Nurses and Care Assistants. London: Health Education England, 2018.
37. Goodwin N, Sonala L, Thiel V, et al. Co-ordinated care for people with complex chronic conditions: Key lessons and markers for success. London: The King's Fund, 2013.
38. NICE. Transition between inpatient hospital settings and community or care home settings for adults with social care needs Nice guideline [NG27] National Institute for Health and Clinical Excellence 2015.
39. Clarke A, Adamson J, Sheard L, et al. Implementing electronic patient record systems (EPRs) into England's acute, mental health and community care trusts: a mixed methods study. *BMC Med Inform Decis Mak* 2015;15:85. doi: 10.1186/s12911-015-0204-0
40. Hillestad R, Bigelow J, Bower A, et al. Can electronic medical record systems transform health care? Potential health benefits, savings, and costs. *Health Aff (Millwood)* 2005;24(5):1103-17. doi: 10.1377/hlthaff.24.5.1103
41. Madden M. Alienating evidence based medicine vs. innovative medical device marketing: a report on the evidence debate at a Wounds conference. *Soc Sci Med* 2012;74(12):2046-52. doi: 10.1016/j.socscimed.2012.02.026
42. Greatbatch. A false economy: Cuts to Continuing Professional Development funding for nursing, midwifery and the Allied Health Professions in England London: Council of Deans of Health, 2016.
43. Ousey K. Reducing funding for CPD courses: what does it mean for tissue viability? . *Wounds UK* 2016;13(1):10-15.
44. Frenk J, Chen L, Bhutta ZA, et al. Health professionals for a new century: transforming education to strengthen health systems in an interdependent world. *Lancet* 2010;376(9756):1923-58. doi: 10.1016/S0140-6736(10)61854-5 [published Online First: 2010/11/30]
45. Rafferty AM, Xyrichis A, Caldwell C. Post-graduate education and career pathways in nursing: a policy brief London: King's College London., 2015.
46. Thompson C, Stapley S. Do educational interventions improve nurses' clinical decision making and judgement? A systematic review. *Int J Nurs Stud* 2011;48(7):881-93. doi: 10.1016/j.ijnurstu.2010.12.005
47. Werdin F, Tennenhaus M, Schaller HE, et al. Evidence-based management strategies for treatment of chronic wounds. *Eplasty* 2009;9:e19.
48. Dumville JC, Deshpande S, O'Meara S, et al. Foam dressings for healing diabetic foot ulcers. *Cochrane Database Syst Rev* 2013(6):CD009111. doi: 10.1002/14651858.CD009111.pub3
49. Dumville JC, O'Meara S, Deshpande S, et al. Hydrogel dressings for healing diabetic foot ulcers. *Cochrane Database Syst Rev* 2013(7):CD009101. doi: 10.1002/14651858.CD009101.pub3
50. Dumville JC, Stubbs N, Keogh SJ, et al. Hydrogel dressings for treating pressure ulcers. *Cochrane Database Syst Rev* 2015(2):CD011226. doi: 10.1002/14651858.CD011226.pub2
51. Forster R, Pagnamenta F. Dressings and topical agents for arterial leg ulcers. *Cochrane Database Syst Rev* 2015(6):CD001836. doi: 10.1002/14651858.CD001836.pub3
52. Jull AB, Cullum N, Dumville JC, et al. Honey as a topical treatment for wounds. *Cochrane Database Syst Rev* 2015(3):CD005083. doi: 10.1002/14651858.CD005083.pub4

For peer review only - http://bmjopen.bmj.com/site/about/guidelines.xhtml

53. O'Meara S, Martyn-St James M. Foam dressings for venous leg ulcers. *Cochrane Database Syst Rev* 2013(5):CD009907. doi: 10.1002/14651858.CD009907.pub2
54. Health Service Executive. National best practice and evidence based guidelines for wound managment. Dublin, Ireland: Health Service Executive, 2009.
55. Boaz A, Hanney S, Jones T, et al. Does the engagement of clinicians and organisations in research improve healthcare performance: a three-stage review. *BMJ Open* 2015;5(12):e009415. doi: 10.1136/bmjopen-2015-009415 [published Online First: 2015/12/15]
56. Luker KA, Kenrick M. An exploratory study of the sources of influence on the clinical decisions of community nurses. *J Adv Nurs* 1992;17(4):457-66.
57. McCaughan D, Thompson C, Cullum N, et al. Nurse practitioner and practice nurses' use of research information in clinical decision making: findings from an exploratory study. *Fam Pract* 2005;22(5):490-7. doi: 10.1093/fampra/cmi042
58. O'Leary D F, Mhaolrunaigh SN. Information-seeking behaviour of nurses: where is information sought and what processes are followed? *J Adv Nurs* 2012;68(2):379-90. doi: 10.1111/j.1365-2648.2011.05750.x
59. Robertson R, Wenzel L, Thompson J, et al. Understanding NHS financial pressures How are they affecting patient care? London: The King's Fund, 2017.
60. Jackson C. Implementing a District and Community Nursing workload tool, to determine safe staffing levels and skill mix in a community care provider organisation: An economic assessment of potential benefits for workforce planning. Kent: Canterbury Christ Church University 2016.
61. Saltman RB. The impact of slow economic growth on health sector reform: a cross-national perspective. *Health Econ Policy Law* 2018:1-24. doi: 10.1017/S1744133117000445
62. Karanikolos M, Mladovsky P, Cylus J, et al. Financial crisis, austerity, and health in Europe. *Lancet* 2013;381(9874):1323-31. doi: 10.1016/S0140-6736(13)60102-6
63. Bussieres AE, Patey AM, Francis JJ, et al. Identifying factors likely to influence compliance with diagnostic imaging guideline recommendations for spine disorders among chiropractors in North America: a focus group study using the Theoretical Domains Framework. *Implement Sci* 2012;7:82. doi: 10.1186/1748-5908-7-82

For peer review only

For peer review only - http://bmjopen.bmj.com/site/about/guidelines.xhtml

Funding

This project was funded by the National Institute for Health Research Collaboration for Leadership in Applied Health Research and Care (NIHR CLAHRC) Greater Manchester. The NIHR CLAHRC Greater Manchester is a partnership between providers and commissioners from the NHS, industry and the third sector, as well as clinical and research staff from the University of Manchester. The views expressed in this article are those of the authors and not necessarily those of the NHS, NIHR or the Department of Health.

Contributors

NC, JD and TG conceived the idea and design for the overall project, PW contributed to further development of the study design. TG, PW and JD collected the data. TG and PW were responsible for data analyses. TG created the original draft of the manuscript. All authors contributed to the interpretation of study findings, critical revision of the manuscript for important intellectual content and approval of the final manuscript.

Acknowledgements

The authors are grateful to many colleagues within the NIHR CLAHRC Greater Manchester Wound Care Programme, who have assisted with and supported this work. We thank all participants for giving their time, sharing their views and for their enthusiasm throughout

Conflicts of interest

None declared

Data sharing statement

Requests for access to data should be addressed to the corresponding author.

For peer review only

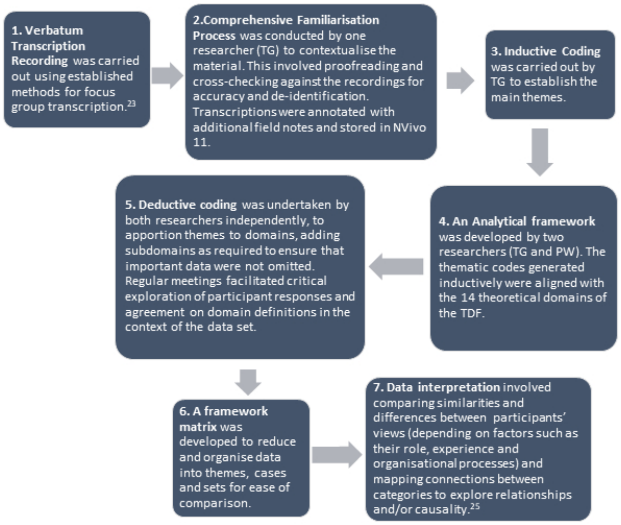

Figure 1. Qualitative analysis using a seven step framework method

254x190mm (96 x 96 DPI)

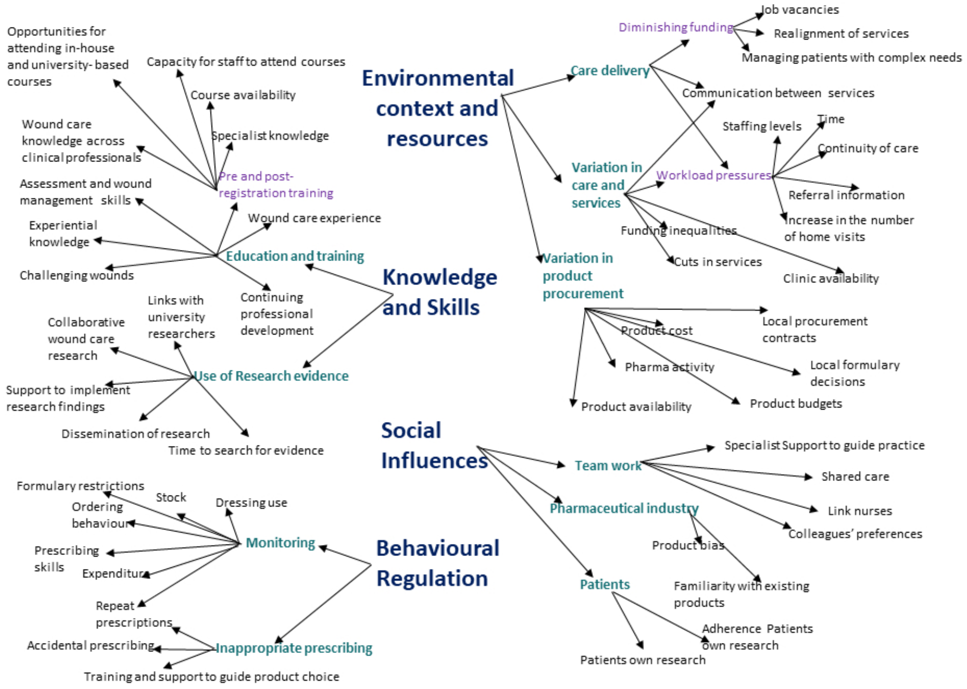

Figure 2. Coding Tree showing the four salient domains with connected sub themes

254x190mm (96 x 96 DPI)

The University of Manchester

National Institute for Health Research

Appendix 1: What Factors Influence Community Wound Care: Clinical Professional Focus Group Questions

Questions	Prompts for further exploration	TDF Domains
How do you decide which dressing or treatment to use for which patient?	What factors contribute and how is it/are they obtained? Please provide examples to explain answers<br>• Knowledge and skills,<br>○ Under/post grad training/regular updates<br>○ Peers/networking<br>○ Experience/Expertise<br>○ Preferences<br>○ Specialist support<br>○ Conferences/seminars<br>○ Pharmaceutical reps/fact sheets<br>• Research evidence<br>○ Reading journals<br>○ On-line search<br>○ National guidelines<br>○ Communicated via wound care specialists<br>• Patient and carers' influence<br>○ Lifestyle<br>○ Adherence<br>○ Choice/Preference<br>○ Anatomical factors /dressing suitability for foot in mobile patient	Skills Knowledge<br>Social professional role and identity<br>Behavioral Regulation<br>Environmental context and resources<br>Beliefs about capabilities<br>Memory, attention and decision processes<br>Beliefs about consequences<br>Motivation and goals<br>Emotion
Are there any environmental (organisational or resource based) factors that influence your prescribing practice?	Are the following enablers or barriers?<br>• Processes e.g. having a formulary in place?<br>○ If a formulary is in place is ordering from it mandatory?<br>• Product cost?<br>• Value for money?<br>○ Are some products worth paying more for e.g. silver/soft silicon?<br>○ What additional benefits do they provide?<br>○ How do you justify the additional cost?<br>• Product availability?<br>• Product knowledge? Why choose one product over another?r<br>• Memory (considering the number of products available)?<br>• Training? Competence?<br>• Caseload? Automony?<br>• Team support?	Behavioral Regulation<br>Environmental context and resources<br>Knowledge<br>Skills<br>Beliefs about capabilities<br>Memory, attention and decision processes<br>Beliefs about capabilities

For peer review only

MANCHESTER 1824
The University of Manchester

NHS
National Institute for Health Research

Questions	Prompts for further exploration	TDF Domains
Do you have any influence on what products are included in your Trust formulary?	How?<br><br>Who else is involved?<br><br>If not you – who decides	Social professional role and identity<br><br>Skills<br><br>Environmental context and resources<br><br>Beliefs about capabilities<br><br>Nature of the behaviors
Have other people or situations ever caused you to change your wound care practices?	An incident? What happened?<br><br>Service reconfiguration? Why was this necessary?<br><br>A change in policy? Why was the policy changed? How was the change implemented?<br><br>A colleague? An expert in the field?<br><br>What processes are in place to share practice relating to product usage?<br><br>Do current networks adequately promote shared cared between teams and services? If not what do you think needs to be done to improve this?	Behavioral Regulation<br><br>Environmental context and resources<br><br>Nature of the behaviors<br><br>Social influences
How do you know you are/your service or your trust is making the right decisions regarding product use and service delivery?	Have you completed any audits of clinical care, clinical outcomes or service outcomes?<br>• Are prescribing practices audited? If so how frequently?<br>• Are prescribing skills audited? If so how frequently?<br>• Are prescribing skills regularly monitored/ appraised? If so how frequently?<br>• Do you have a PDP? Do attend regular personal development reviews? How often?<br>• Do you receive regular updates at trust or service level regarding service delivery achievements?<br>What other measures are in place to monitor prescribing practices?	Behavioral Regulation<br><br>Motivation and goals

For peer review only

The University of Manchester

National Institute for Health Research

Now you have had time to look at the wound care product expenditure………..		
**Questions**	**Prompts for further exploration**	**TDF Domains**
Are the overall figures what you were expecting?	Is the overall spend higher or lower than you were expecting?<br><br>What do you think has caused the difference (if any)?<br><br>How do you feel about the differences or similarities with neighboring Trusts and national figures?	Knowledge<br><br>Beliefs about consequences<br><br>Behavioral Regulation<br><br>Environmental context and resources
Does the expenditure for any particular product group surprise you?	Are you surprised that it is low or high expenditure?<br><br>Why does it surprise you?<br><br>What do you think has caused this?<br><br>How do you feel about the differences with neighboring Trusts and national figures?	Knowledge<br><br>Beliefs about consequences<br><br>Environmental context and resources<br><br>Behavioral Regulation
Do you think there is over or under use of any product group?	Which group(s)? Is this over or under used?<br><br>What do you think has caused this?<br><br>Prior to seeing the figures, which product groups did you believe were used the most frequently?<br><br>Prior to seeing the figures, which product groups did you believe were used the least frequently?<br><br>How do you feel about the comparison with neighboring Trusts and national figures?	Knowledge<br><br>Skills<br><br>Beliefs about consequences<br><br>Environmental context and resources

The University of Manchester

National Institute for Health Research

Appendix 2. What Factors Influence Community Wound Care: Non-clinical Professional Focus Group Questions

Introductory statement to clarify the focus: As you all represent different organisations and have different roles, our questions will aim to establish the procurement processes and practices across four community healthcare organisations (that have partnered with CLAHRC GM to deliver this work) and neighbouring partner CCGs. When we say 'local' we are referring to the 14 services involved. We will use prompts to explore each question further so that we have a clear understanding of each of the services involved.

Questions	Prompts for further exploration	TDF Domains
What procurement processes are in place locally?	Stock list only? Prescribing only? Combination?<br><br>How is information about the systems/practices circulated to community and primary care staff? Are communication channels monitored?<br><br>Do Trusts/CCGs have a formulary?<br>• How are they compiled/updated?<br>• Who is involved?<br>• Who influences what is included?<br><br>What are the drivers for changing processes/practice? Do you have examples?<br>• Incidents?<br>• Audit findings?<br>• Service reconfiguration?<br>• Research evidence?<br>• Lessons learnt from other services?	Environmental context and resources<br><br>Knowledge<br><br>Skills<br><br>Social professional role and identity<br><br>Social influences

For peer review only

The University of Manchester

National Institute for Health Research

Questions	Prompts for further exploration	TDF Domains
Are there any factors that influence procurement decisions?	Are the following enablers or barriers?<br>• Product cost?<br>• Product availability?<br>• Product knowledge?<br>• Memory (considering the number of products available)?<br>• Training?<br>• Competence?<br>• Team support?<br>• Company reps<br>○ access monitored/unmonitored<br>○ Incentives	Behavioral Regulation<br><br>Environmental context and resources<br><br>Knowledge<br><br>Skills<br><br>Beliefs about capabilities<br><br>Memory, attention and decision processes
What locally agreed CQUINs/policies are in place for wound care management?	What do these entail?<br><br>Are there any requirements to conduct audits to monitor adherence to agreed wound care management pathway?<br>• Six monthly/yearly?<br>• What are the consequences for poor adherence<br><br>Do they include incentive schemes?<br>Does they include a commitments to reduce spend e.g. reduce silver dressings spend.<br>Do these include a commitment to undertake an ongoing programme of educational training to community or primary care staff on agreed wound management pathway (inc formulary)?<br><br>Do they include a commitment to benchmark with other organisations? (Which?)	Behavioral Regulation<br><br>Environmental context and resources<br><br>Beliefs about consequences<br><br>Motivation and goals<br>Skills
Do healthcare professionals follow policy when ordering /prescribing wound care products?	What percentage of products are ordered/prescribed 'off formulary'<br><br>Are there specific products that are prescribed regularly 'off formulary'?<br><br>Are there any incentives to promote good prescribing/ordering practices?<br><br>If a stock list exists, are stock products sometimes prescribed rather than ordered from the stock list?<br><br>What are the consequences of not following policy/ordering off formulary?<br><br>What do you think can be done to improve adherence to policy?	Behavioral Regulation<br><br>Environmental context and resources<br><br>Beliefs about consequences

For peer review only

The University of Manchester

National Institute for Health Research

Now you have had time to look at the wound care product expenditure………..		
**Questions**	**Prompts for further exploration**	**TDF Domains**
How do you feel about the differences or similarities in expenditure locally compared to the national spend?	Is the overall spend higher or lower than you would like it to be?<br><br>How do you feel about the differences or similarities with the rest of GM and the national figures?<br><br>If overall spend is higher than average what do you think can be done to reduce the spend?	Knowledge<br><br>Beliefs about consequences<br><br>Behavioral Regulation<br><br>Environmental context and resources
Is the expenditure for any particular product higher or lower than you think it should be?	Why do to think expenditure is higher or lower?<br><br>Which type of HCP is contributing to the high/low expenditure?<br><br>How does this compares with the rest of the region and the national figures?	Knowledge<br><br>Beliefs about consequences<br><br>Environmental context and resources<br><br>Behavioral Regulation
Do you think there is over or under use of any product group?	Which group(s)? Is this over or under use?<br><br>What do you think has caused this?<br><br>How does this compare with regional and the national figures?	Knowledge<br><br>Skills<br><br>Beliefs about consequences<br><br>Environmental context and resources

For peer review only - http://bmjopen.bmj.com/site/about/guidelines.xhtml

Item No	Topic	Guide Questions/Descriptions	Reported on Page No (clean copy)
**Domain 1 Research Team and reflexivity**			
Personal Characteristics			
1	Interviewer/facilitator	Which author/s conducted the interview or focus group?	8
2	Credentials	What were the researcher's Credentials E.g. PhD, MD Occupation	8
3	Occupation	What was their occupation at the time of the study?	8
4	Gender	Was the researcher male or female?	8
5	Experience and training	What experience or training did the researcher have?	8
6	Relationship established	Was a relationship established prior to study commencement?	7
7	Participant knowledge of the interviewer	What did the participants know about the researcher? e.g. personal goals, reasons for doing the research	7
8	Interviewer characteristics	What characteristics were reported about the interviewer/facilitator? e.g. Bias, assumptions, reasons and interests in the research topic	8
**Domain 2: Study design**			
Theoretical framework			
9	Methodological orientation and Theory	What methodological orientation was stated to underpin the study? e.g. grounded theory, discourse analysis, ethnography, phenomenology, content analysis	5/9
10	Sampling	How were participants selected? e.g. purposive, convenience, consecutive, snowball	7
11	Method of approach	How were participants approached? e.g. face-to-face, telephone, mail, email	7
12	Sample size	How many participants were in the study?	7/9/10
13	Non-participation	How many people refused to participate or dropped out? Reasons?	9
	Setting		
14	Setting of data collection	Where was the data collected? e.g. home, clinic, workplace	7
15	Presence of nonparticipants	Was anyone else present besides the participants and researchers?	8
16	Description of sample	What are the important characteristics of the sample? e.g. demographic data, date	8/11
	Data collection		
	Interview guide	Were questions, prompts, guides provided by the authors? Was it pilot tested?	Appendix 1and 2/8
	Repeat interviews	Were repeat inter views carried out? If yes, how many?	N/A
	Audio/visual recording	Did the research use audio or visual recording to collect the data?	8
	Field notes	Were field notes made during and/or after the interview or focus group?	8

For peer review only

	Duration	What was the duration of the inter views or focus group?	9
	Data saturation	Was data saturation discussed?	7
	Transcripts returned	Were transcripts returned to participants for comment and/or correction?	8
**Domain 3: analysis and findings**			
	Data analysis		
	Number of data coders	How many data coders coded the data?	Figure 1
	Description of the coding tree	Did authors provide a description of the coding tree?	Figure 2
	Derivation of themes	Were themes identified in advance or derived from the data?	8/Figure 1
	Software	What software, if applicable, was used to manage the data?	9
	Participant checking	Did participants provide feedback on the findings?	8
	Reporting		
	Quotations presented	Were participant quotations presented to illustrate the themes/findings? Was each quotation identified? e.g. participant number	11-15
	Data and findings consistent	Was there consistency between the data presented and the findings?	10-15
	Clarity of major themes	Were major themes clearly presented in the findings?	10-15
	Clarity of minor themes	Is there a description of diverse cases or discussion of minor themes?	10

Developed from: Tong A, Sainsbury P, Craig J. Consolidated criteria for reporting qualitative research (COREQ): a 32-item checklist for interviews and focus groups. International Journal for Quality in Health Care. 2007. Volume 19, Number 6: pp. 349 – 357

For peer review only

BMJ Open

WHAT FACTORS INFLUENCE COMMUNITY WOUND CARE IN THE UK? A focus group study using the Theoretical Domains Framework

Journal:	*BMJ Open*
Manuscript ID	bmjopen-2018-024859.R2
Article Type:	Research
Date Submitted by the Author:	04-Jun-2019
Complete List of Authors:	Gray, Trish; University of Manchester, Division of Nursing, Midwifery and Social Work, School of Health Sciences, Faculty of Biology, Medicine and Health<br>Wilson, Paul; University of Manchester, Alliance Manchester Business School<br>Dumville, Jo C.; University of Manchester, Division of Nursing, Midwifery and Social Work, School of Health Sciences, Faculty of Biology, Medicine and Health<br>Cullum, Nicky; The University of Manchester, Division of Nursing, Midwifery and Social Work, School of Health Sciences, Faculty of Biology, Medicine and Health
**Primary Subject Heading**:	Qualitative research
Secondary Subject Heading:	Health services research
Keywords:	QUALITATIVE RESEARCH, FOCUS GROUP, WOUND MANAGEMENT, HEALTHCARE PROFESSIONAL, HEALTHCARE QUALITY, THEORETICAL DOMAINS FRAMEWORK

SCHOLARONE™ Manuscripts

WHAT FACTORS INFLUENCE COMMUNITY WOUND CARE IN THE UK? A focus group study using the Theoretical Domains Framework

Corresponding Author

Trish Gray
Division of Nursing, Midwifery and Social Work; School of Health Sciences;
Faculty of Biology, Medicine and Health.
The University of Manchester, Room 6.332, Jean McFarlane Building,
Oxford Road, Manchester, M13 9PL.
Email: Trish.Gray@manchester.ac.uk
Tel: (+44) (0)161 306 7849

For peer review only

Authors:

Trish A Gray,[1,3.] Paul Wilson,[2,3] Jo C Dumville,[1,3] Nicky A Cullum.[1,3,4]

Affiliations

1 Division of Nursing, Midwifery and Social Work, School of Health Sciences, Faculty of Biology, Medicine and Health, University of Manchester, Manchester, UK

2 Alliance Manchester Business School, University of Manchester, Manchester, UK

3 NIHR CLAHRC Greater Manchester 3rd Floor Mayo Building, Salford Royal NHS Foundation Trust, Stott Lane, Salford, Manchester, UK

4 Research and Innovation Division, Central Manchester University Hospitals NHS Foundation Trust, Grafton Street, Manchester, UK

Word Count

Abstract 289
Main text: 4488

KEY WORDS

Qualitative research, focus group, wound management, healthcare professional, healthcare quality, Theoretical Domains Framework.

For peer review only - http://bmjopen.bmj.com/site/about/guidelines.xhtml

ABSTRACT:

Objectives: Research has found unwarranted variation across community wound care services in the North of England, with underuse of evidence-based practice and overuse of interventions where there is little or no known patient benefit. This study explored the factors that influence care in community settings for people with complex wounds to develop a deeper understanding of the current context of wound care and variation in practice.

Design: Qualitative focus group study using The Theoretical Domains Framework to structure the questions, prompts and analyses.

Setting: Community healthcare settings in the North of England, UK.

Participants: Forty-six clinical professionals who cared for patients with complex wounds and eight non-clinical professionals who were responsible for procuring wound care products participated across six focus group interviews.

Results: We found the TDF domains: Environmental context and resources, Knowledge, Skills, Social influences and Behaviour regulation to best explain the variation in wound care and the underuse of research evidence. Factors such as financial pressures were perceived as having a negative effect on the continuity of care, the availability of wound care services and workloads. We found practice to be mainly based on experiential knowledge and personal preference and highly influenced by colleagues, patients and the pharmaceutical industry though not by research evidence.

Conclusions: Our study provides new insight into the role that experiential learning and social influences play in determining wound care and on the limited influence of research. Workforce pressures and limited resources are perceived to impede care by reducing patient access to services and the ability to provide holistic care. Participative collaboration between university and healthcare organisations may offer a supportive route to addressing issues, implementing sustainable changes to practice and service delivery and a resolute commitment to research use amongst clinical professionals.

For peer review only

For peer review only - http://bmjopen.bmj.com/site/about/guidelines.xhtml

Article Summary

Strengths and Limitations of this study

- This focus group study is the first to explore the factors that influence wound care and the reasons for known variation in practice.
- Employing a qualitative methodology provided new insight into the role experiential learning and social influences play in determining clinical and procurement choices.
- The focus group design stimulated discussion allowing participants to examine their own and others' views and experiences.
- The Theoretical Domains Framework provided a theoretical structure for developing a deeper understanding of wound care delivery.
- The sample was taken from community healthcare organisations in the North of England, inclusion of participants from a larger geographical population may have provided different views.

MAIN TEXT

Introduction

People with complex wounds (open wounds, such as foot, leg and pressure ulcers, burns, open trauma and surgical wounds that are difficult to heal),[1 2] are more likely to be elderly and living with multimorbidity.[3] In the UK, the management of people with complex wounds[1 2] is mainly carried out in patients' homes or community clinics by community nurses with advice and support from specialist teams (nurses and medics with expertise in tissue viability, burns, vascular medicine or dermatology). Podiatrists also play a vital role in managing complex foot wounds, often working in conjunction with community nurses.

Care of complex wounds in community settings normally includes a comprehensive assessment of the person and their wound (involving demographics, risk factors for wound healing, quality of life measures, wound status,

For peer review only - http://bmjopen.bmj.com/site/about/guidelines.xhtml

wound parameters and symptoms), specific wound-related assessments such as ankle brachial pressure index (ABPI) for people with venous leg ulcers and implementation of appropriate interventions.[4] Interventions may involve wound cleansing followed by dressing to manage exudate and protect the wound. Whilst dressings are used widely across wound types, with many different options available for use, there is currently no evidence that one dressing type is more clinically or cost effective than another, even in the case of relatively expensive anti-microbial dressings. In contrast there are effective first line treatments which should be widely used, such as the use of compression therapy for venous leg ulceration which is known to reduce time to wound healing[5 6]

As part of a wider programme of wound care research funded by the National Institute for Health Research Collaboration for Leadership in Applied Health Research and Care (NIHR CLAHRC) Greater Manchester, we conducted a survey to assess how healthcare professionals managed wound care across five community healthcare organisations in the North of England.[7] The findings are discussed in more detail elsewhere[7] but in summary the survey revealed unwarranted variation in clinical practice, with general underuse of Doppler-aided measurement of ABPI,[8] underuse of compression therapy and[9] potential overuse of antimicrobial dressings.[6] In the UK, variations in wound care are being recognised and addressed with initiatives such the Leading Change, Adding Value Nursing and Midwifery Framework[10 11] however, there has been little formal exploration of drivers for this variation in the delivery of wound care and barriers to implementing the findings from current research evidence. In turn there is little intelligence to guide further research implementation and bring about meaningful practice change with the aim of maximising patient benefit.

Our aim was to identify and explore factors that influence care in community settings for people with complex wounds. We wanted to better understand the current context of community wound care and how research evidence informs care delivery.

For peer review only - http://bmjopen.bmj.com/site/about/guidelines.xhtml

Methods

Design

We conducted six focus group interviews to explore the factors that influence the care of people with complex wounds in community settings. The Theoretical Domains Framework (TDF) was used to structure the questions, prompts and analyses.[12 13] The TDF provides a theoretical lens through which to view cognitive, affective, social and environmental factors that could potentially influence behaviour.[14] It has been used extensively across a range of clinical areas.[15-17] Its constructs are grouped into 14 discrete domains.[12] The TDF is presented in Table 1 showing the domains, definitions and examples of behaviours related to wound care and wound product procurement.

For peer review only

For peer review only - http://bmjopen.bmj.com/site/about/guidelines.xhtml

Table 1: The Theoretical Domains Framework: domains, definitions and examples of behaviours related to wound care and wound product procurement

Domain	Definition	Examples of wound care and wound product procurement behaviors
Knowledge	An awareness of the existence of something.	Knowledge of wound types, wound aetiology, risk factors, wound product types and influenced by education, experience, research
Skills	An ability or proficiency acquired through practice.	Ability to complete a comprehensive wound assessment, specific assessments such as ABPI, apply compression bandages/stockings, managing procurement processes effectively
Social/Professional role and identity	A coherent set of behaviors and displayed personal qualities of an individual in a social or work setting.	Carrying out a clinical or procurement role according to job description, communicating and working appropriately and effectively with other clinical or non-clinical professionals
Beliefs about capabilities	Acceptance of the truth, reality, or validity about an ability, talent, or facility that a person can put to constructive use.	Being confident in making the right decisions about care for patients with complex wounds, confidence in negotiating skills for product procurement
Optimism	The confidence that things will happen for the best or that desired goals will be attained.	Confidence that care provided will achieve cure/manage wounds effectively, confident that most cost effective products can be purchased
Beliefs about consequences	Acceptance of the truth, reality, or validity about outcomes of a behavior in a given situation.	Having realistic views about patient adherence to treatment plans and healing rates of complex wounds
Reinforcement	Increasing the probability of a response by arranging a dependent relationship, or contingency, between the response and a given stimulus.	Support of colleagues, team work, wound care provided has produced the desired goal, research evidence that interventions work
Intentions	A conscious decision to perform a behaviour or a resolve to act in a certain way.	To practice according to a care plan, national/international guidelines
Goals	Mental representations of outcomes or end states that an individual wants to achieve.	Setting goals for wound healing, improving patient adherence, achieving competence for a new skill
Memory, attention and decision processes	The ability to retain information, focus selectively on aspects of the environment and choose between two or more alternatives.	Ability to remember wound care information learnt, dressing specifications considering the wide choice, making decisions based on evidence
Environmental context and resources	Any circumstance of a person's situation or environment that discourages or encourages the development of skills and abilities, independence, social competence and adaptive behavior.	Organisational structures, procedures and processes, workload pressures, staff shortages, funding constraints, service cuts, procurement processes, product cost, product availability,
Social influences	Those interpersonal processes that can cause individuals to change their thoughts, feelings, or behaviors.	Decisions influences by personal, colleagues' patients, pharmaceutical industry preferences, team work and shared care, understanding patients' needs, negotiating product cost
Emotion	A complex reaction pattern, involving experiential, behavioral, and psychological elements, by which individual attempts to deal with a personally significant matter or event.	Coping with wounds that do not heal, managing challenging wounds, dealing with emotions when patients with complex needs deteriorate or die
Behavioral regulation	Anything aimed at managing or changing objectively observed or measured actions.	Formulary to guide (restrict) prescribing and procurement choices, audits of practice and procedures

123 Adapted from © Cane et al.; licensee BioMed Central Ltd.

For peer review only

For peer review only - http://bmjopen.bmj.com/site/about/guidelines.xhtml

Participants and settings

Purposive sampling was used to ensure that we recruited participants with relevant clinical and/or procurement experience. Eligibility included community-based clinical professionals who cared for patients with complex wounds or non-clinical professionals who were involved in the procurement of wound care products. Clinical professionals included community nurses, podiatrists, tissue viability or burns specialist nurses, wound research nurses and clinical nurse managers (who had a clinical role, managed a team of community nurses and were responsible for wound product procurement decisions). Non-clinical professionals included: medicines optimisation pharmacists, procurement leads, procurement advisors and medicines management leads. There were five multidisciplinary focus group interviews for clinical professionals; one for each participating provider organisation. Four were drawn from provider organisations in one defined geographical area with a fifth conducted in a different geographical area but similar urban conurbation in the North of England; chosen for its well-established links with university researchers as a comparison to the other organisations where collaborative partnerships with university researchers were in their infancy. A separate focus group interview was held for non-clinical professionals. As the themes for clinical and non-clinical focus group interviews differed, we chose to separate clinical from non-clinical professionals to maintain focus and create an optimum environment for free flowing discussions. Potential participants were identified through contacts developed as part of the NIHR CLAHRC GM wound care programme and were approached via email, telephone or face-to-face meeting. Focus group interviews were held locally to participants' work place in a healthcare setting or conference centre.

For peer review only

As participants were drawn from a relatively homogeneous population and the interview schedules were focused on specific aspects of wound care and wound product procurement, we anticipated that we would reach data saturation within three to four focus group interviews, however, to incorporate all partner provider organisations using the format described above we needed to recruit 50-60 participants in total across the six groups (to allow for 8 to 10 participants per group), based on recommendations from existing literature.[18-21]

For peer review only - http://bmjopen.bmj.com/site/about/guidelines.xhtml

Data collection

The format was similar for all focus group interviews; they were facilitated by a lead (TG) with one or two co-facilitators (PW and JD). All facilitators were experienced researchers and familiar with the evidence base for wound care. A fourth member of the research team took field notes. Before the session began, participants were asked to complete a brief demographic questionnaire to clarify their academic and professional qualifications and wound care/product procurement experience as relevant; these data were used to describe the participants involved and were not linked to particular responses or quotes. Each session was audio recorded with recordings deleted following verification of anonymised transcripts.

Procedure

The discussion explored specific behaviours linked to the TDF domains and reactions to site-specific, regional and national procurement data using the questions and prompts outlined in Appendix 1. Clinical professionals were encouraged to think about factors that from their experience, enable or hinder the delivery of wound care, relating their answers to their own experiences. Through prompts we probed further, allowing participants' reactions to unfold, giving them the opportunity to explore their own and others' views. We continued to prompt if responses were not spontaneously offered to encourage full participant engagement. The focus group interview for non-clinical professionals followed an identical format with the questions more related to procurement systems and procedures

(Appendix 2). Interview schedules were piloted by specialist nurses, clinical managers and a procurement lead, after which minor amendments were made. Respondents validated the accuracy and completeness of the findings[22] following a verbal summary (taken from the field notes) at the end of each focus group interview and a post-analysis report sent via email.

For peer review only - http://bmjopen.bmj.com/site/about/guidelines.xhtml

Ethical considerations

Ethics approval was sought and granted from the University of Manchester Research Ethics Committee (Refs 15272, 15327 and 2017-0559-1767) and HRA approval was sought and granted (Refs IRAS 174691, 184865 and 219918). Written informed consent was obtained from all participants.

Patient and Public Involvement

Views expressed by members of the NIHR CLAHRC Greater Manchester Wounds Research PPI Forum about their experiences with healthcare professionals and wound care services were used to inform some of the questions and prompts for the focus group interviews.

Data Analysis

Quantitative data were stored in SPSS (IBM version 22). Demographic variables are expressed in frequencies, means and standard deviations where distributions are normal, and medians and range when skewed. Qualitative analysis followed a seven-step process in line with the framework method (Figure 1).[23-25]

Findings

Participant characteristics

Sixty participants were invited to attend one of six focus group interviews (mean duration: 106 minutes). Fifty-four participants attended whilst nine invited participants could not attend due to other clinical commitments or annual leave (three of whom nominated colleagues to attend in their place). Participants comprised 46 clinical professionals (ten specialist nurses (19%), 25 community nurses (46%), seven podiatrists (13%), three clinical managers (5%) and one research nurse (2%)) and eight non-clinical professionals (15%). Wound care experience was extensive (mean 14.6 years, SD 8.8) amongst clinical professionals (Table 2).

For peer review only

For peer review only - http://bmjopen.bmj.com/site/about/guidelines.xhtml

Table 2. Participant Characteristics (n=54)	N (%)
Gender	
Male	7 (13)
Female	47 (87)
Role group Clinical professional	
Specialist nurse	10 (19)
Community nurse	25 (46)
Research nurse	1 (2)
Clinical Manager	3 (5)
Podiatrist	7 (13)
**Role group *Non-clinical professional***	8 (15)
Highest Academic Qualification	
MSc	6 (11)
BSc/BA (Hons)	27 (50)
PG Diploma	11 (20)
PG Certificate	2 (4)
Vocational Qualification	5 (9)
A level	3 (6)
Years in current role	**Mean (SD)**
Clinical professional	8.6 (7.4)
Non-clinical professional	4.7 (4.3)
Years of wounds care/procurement experience	
Clinical professional	14.5 (8.8)
Non-clinical professional	5.7 (6.4)
Attended a wound care update in last 12 months (n=46)	***N (%)***
Yes	15 (33)
No	31 (67)
Attended wound procurement update in last 12 months (n=8)	
Yes	1 (13)
No	7 (88)

198

199 Key themes identified within relevant domains

200 Five TDF domains dominated: *Environmental context and resources*, *Knowledge, Skills, Social influences* and
201 *Behaviour regulation*. The domains of knowledge and skills were closely linked and frequently overlapped,
202 therefore, we combined these. We did not code any source data to the domains of *Emotion* and *Intentions* and
203 found the remaining six domains to overlap with the five dominant domains. We have therefore, focused on
204 the five key domains which best explain the variation in wound care and the underuse of research evidence.
205 The coding tree (Figure 2) demonstrates the relationships between domains and sub-themes.

For peer review only

Environmental context and resources

Delivery of care

Clinical professionals across all groups expressed feeling the pressure of increased workloads. Some participants said they were working more intensely and without breaks, constantly feeling anxious that they may have missed something as time was limited between patient consultations. They reported that there was an increase in sick leave, experienced colleagues were leaving and their roles were left vacant.

"You haven't got the same skill base any more. We haven't got the same expertise, we're losing our experienced link nurse this week, and we haven't really got anybody with that level of skill in wounds to take her place........we've got 30 vacancies at the moment that haven't been filled" (Clinical manager)

Community nurses reported that specialist clinics were being cut, and patients previously seen in dedicated leg ulcer clinics by nurses with specialist knowledge, were now visited at home by understaffed community nursing teams.

"Physically running the clinic was based on when there was about six or seven [leg ulcer specialist] staff ... when it was a leg ulcer service. There's only two of us so we haven't got the capacity to cover those let alone do all the home visits." (Community nurse)

Community nurses and podiatrists voiced concern that undue time was spent gathering required patient information due to poor referral information supplied by hospital staff.

"You constantly are ringing because they'll [ward staff] *put* [on the referral] *"care of wound", but …… what wound have they got? What operation have they had? What would you like me to do with it? It's very, very poor."* (Community nurse)

Variation in care and services

Many clinical participants attributed variation in the patterns of care delivery to realignment of services due to reduced funds. The majority of clinical professionals reported that specialist leg ulcer clinics had been cut resulting in a greater number of home visits for community nurses. Participants from the research active organisation, reported that practice nurses (nurses based in a general practitioner practice providing primary care for a local population) managed mobile patients with wounds, whilst community nurses cared for

For peer review only

housebound patients with more complex health needs. This changed model of service delivery was felt by the community nurses to have eased their workload.

Participants from organisations that managed both hospital (acute) and community services felt that resourcing prioritised the acute service at the expense of the community service. Participants made reference to the differences between resources available in acute care that were limited or unavailable in the community; this included wound care products and digital technology.

"I just don't feel the acute side has got a grip at all on community services in terms of what we do…I mean, I do a specialist [acute] clinic *on a Tuesday morning and have access to all sorts of dressings. And I come back into the community….and we're very limited, we've got one foam* [dressing] *that we can use*." (Podiatrist)

Clinical participants viewed access to photographic equipment as a valuable resource that allowed images of wounds to be sent to a podiatrist or specialist nurse for rapid diagnosis and care planning, however, only healthcare professionals with access to hospital photographic equipment could make use of this service. One specialist nurse apologised for using photographic equipment that the community nurses within her organisation did not have access to.

"I do [take photographs of wounds]. I've got a camera. Sorry. It is downloaded onto a programme at the hospital. So that's probably why [I have access to it]." (Specialist nurse)

Variation in product procurement

Participants reported a variety of wound care product procurement processes; some (across two provider organisations) obtained all products via prescription, others (across two provider organisations) used a combination of prescribing and stock purchase and one group (one organisation) operated a total stock purchase system. All participants noted the local use of wound care formularies (a locally developed list of recommended products), to guide prescribing or purchasing decisions,[26] however, through discussion it was recognised that the products listed and the number of product available varied across formularies. One organisation had a very restrictive formulary and monitored use closely; participants found this restrictive formulary enabled them to choose appropriate products.

For peer review only - http://bmjopen.bmj.com/site/about/guidelines.xhtml

"I think it's an enabler, … there are so many [dressings to choose from] *you can go completely for something that costs so much and something that wouldn't be right … but having that formulary means that we know what we can choose."* (Community nurse*)*

Knowledge and skills

Education and training

All nurse participants agreed that there was a limited amount of wound care education for student nurses and that most wound care knowledge and skills were gained through community placements rather than in the classroom. Only specialist nurses had attended a university-based post-registration wound care course. In contrast, podiatrists received regular undergraduate and postgraduate wound care education. . All clinical professionals viewed wound care knowledge across other services (hospital, primary care and nursing homes) to be poor, which increased their workload if aspects of care, documentation or prescription information were incomplete.

Specialist nurses reported that due to workforce pressures, in-house courses they offered were often cancelled or attendance was poor. Due to these difficulties, specialist nurses relied on the pharmaceutical industry to provide wound product training sessions. Concerns were raised particularly by the non-clinical professionals that the educational contributions of industry representatives were highly likely to favour their own products.

"But then at the same time they'd spy the competition and they'd basically suggest that their products are equivalent to those products that were already on the shelf …….and then we were inundated with requests for new products*."* (Non-clinical professional)

Use of research evidence

Only participants from the provider organisation with a history of collaborative wound care research indicated that they actively sought to keep up to date with research. Specialist nurses from this focus group talked about their established links with university researchers and their involvement in co-producing wounds research with academics. They discussed disseminating relevant research findings through electronic newsletters, workshops and meetings with community staff and where capacity allowed, staff were supported to implement research findings. Participants reported that their organisation was highly research active in wound care; clinical

For peer review only - http://bmjopen.bmj.com/site/about/guidelines.xhtml

professionals had participated in research that found compression stockings to be more cost-effective than compression bandages for people with venous leg ulcers[9] and they subsequently implemented the findings into practice. The remaining participants viewed research with caution, they found very little time to search for evidence or be involved in research.[6 27 28]

"I can't know everything about all dressings, and therefore you often stick to what you know and you don't often have time to look at research." (Non-clinical professional)

"And as healthcare professionals it's not built into our contracts to do research…there's no time put aside." (Specialist nurse)

Social influences

Team work

The importance of good teamwork was frequently emphasised and acknowledged by all participants. Much of the sharing of experiences was conducted informally. Clinical professionals reported that team support alleviated some of the current workload pressures and shared care was viewed as a valuable method for joint decision making. Participants from one focus group only reported the existence of wound care link nurses whose role was to cascade new information, new research evidence and product updates from specialist nurses to their colleagues. However, capacity issues were affecting the scope of this role.

Industry and patient influence

As referred to above, all participants were concerned about the influence that the pharmaceutical industry had on product choices. It was felt that this influence varied depending on how closely pharmaceutical representatives' access was monitored. Non-clinical participants were particularly and negatively vociferous about the influence of pharmaceutical representatives yet viewed the role of policing any promotional activity as a specialist nurse responsibility.

"You can police this a little bit more in acute, can't you, but in the community we were fighting a losing battle with the reps when they're just given free range to provide training."(Non-clinical professional)

For peer review only - http://bmjopen.bmj.com/site/about/guidelines.xhtml

Participants were very aware of the influence that patients have on wound care, which at times caused difficulty finding a suitable dressing that met patients' expectations. Participants reported that some patients removed dressings earlier than necessary if minor staining appeared. Participants were mindful that careful assessment and monitoring patients' adherence to therapy was necessary when making product choices. Participants also found that patients searched for information on the internet in an attempt to influence product decisions.

"She [the patient] *read that honey was good and she thought I'll go and buy my own…. and swore it did the trick, so who are we to argue with her?"* (Community nurse)

Behaviour regulation

Community nurses reported that antimicrobial dressings (particularly silver-impregnated dressings) were used for individual patients for a two-week trial period and then reviewed, however, they acknowledged that if use was not closely monitored there was potential for overuse. Non-clinical professionals and specialist nurses were aware of the high expenditure on antimicrobial dressings but acknowledged difficulties in monitoring effectively and providing adequate training and support due to capacity issues.

"Silver spend is still a problem and it's a long-term……I think there's still habitual use, district nurses having the time to stop and think and review and stop a treatment rather than continue." (Specialist nurse)

Specialist nurses reported that general practitioners regularly prescribed high cost antimicrobial dressings for nursing home residents. The prescription for these dressings would often be repeated without review unless the resident was referred to a specialist nurse. There was an opinion amongst participants that some prescribing of silver dressings may be accidental because dressings are listed alphabetically in some prescribing platforms and silver dressings appear first (as they are denoted by the chemical symbol for silver, 'Ag').

For peer review only - http://bmjopen.bmj.com/site/about/guidelines.xhtml

Discussion

We believe this is the first study to explore factors influencing care in community settings for people with complex wounds whilst seeking to understand the reasons for known variation in practice. Overall, participants described a challenging working environment, with influences such as workforce shortages and diminishing treatment resources having a marked effect on continuity of care, patient access to services and workload. Clinical practice seemed to be predominantly based on experiential knowledge, personnel preference and to be highly influenced by colleagues, patients and the pharmaceutical industry.

Workforce pressures and diminishing resources

Wound care services were described by participants as a working environment characterised by increasing time pressures and diminishing resources. Roles were perceived as becoming task orientated which was felt to dilute the quality of care. Participants reported there was a rise in sickness, colleagues were leaving for less pressured roles and vacancies were not being filled. UK surveys of community nursing services have found similar results.[29-31] The UK has fewer nurses relative to the population than many EU countries.[32] The number of community nurses is falling, with an estimated vacancy rate of 9.4%.[33] Forty percent of experienced nurse positions are vacant.[34] Championing flexible career pathways, integrated care and the introduction of combined hospital and community posts (to standardise practice, improve care coordination and vary work experiences) have been proposed by UK governing bodies to improve retention rates.[35-38]

Participants reported that specialist clinic sessions had been cut, resulting in increasing workload pressures for community nurses. A systematic review of 27 studies found improved information technology, including remote specialist consultations, to improve access to specialist input, provide educational support for the referrer, shorten referral time and avoid unnecessary travel and inappropriate visits.[39]

For peer review only - http://bmjopen.bmj.com/site/about/guidelines.xhtml

Experiential learning and social influences

All nursing participants agreed that there was a lack of wound care education in basic nurse education. Wound care skills were learnt during community but not hospital placements. This was verified by participants' reference to insufficient information from hospital nursing and medical staff on referral forms and via telephone calls which cause delays in assessment and frustration for community nurses. Whilst all specialist nurse teams offered on-going wound care training to community nurses, cancellation or poor attendance frequently occurred due to staff shortages. By contrast, podiatrists' viewed their wound care education to be strong before and after qualification.

In the light of the current workforce issues and the difficulties community nurses had updating their wound care knowledge, other strong influences played a significant role in wound care choices such as personal, colleague and patient preferences as well as the influence of pharmaceutical company representatives. This influence can drive variation in dressing and treatment choice depending on the amount of access pharmaceutical representatives have to healthcare settings and clinical professionals' attitude to the information they provide.[40] The ongoing cuts to continued professional development funding in the UK since 2015 may lead to greater dependence on the pharmaceutical industry for training and 'education' which is problematic due to companies' vested interests in the use of specific products.[41 42] Inter-professional education may break down professional boundaries and provide opportunities for mutual learning and joint solutions across professional groups and specialties.[43] Further investment into evaluated training interventions that are of high quality and independent is warranted to ensure education is consistent and effective; providing healthcare professionals with the confidence to make the right decisions to improve continuity and quality of care.[36 44-46]

For peer review only - http://bmjopen.bmj.com/site/about/guidelines.xhtml

The influence of research on wound care

Using research to guide product choice

There is a plethora of wound products available for use but, as several Cochrane systematic reviews have shown, there is a paucity of research evidence showing that products are clinically effective.[6 47-52] Despite this, product use and expenditure grow; particularly antimicrobial dressing use, where no compelling evidence or guideline recommendations exist to support routine use (Hussey et al., 2018 manuscript submitted for publication).

We found that a restrictive formulary was viewed as enabling better patient management, particularly if guidelines accompanied the formulary. Community nurses found a formulary and guidance gave them more assurance that they were making the right decisions and specialist nurses found formularies reduced inappropriate product choices and assisted in standardising product use across their service. For the majority of organisations, however, the formulary acted as guidance only and 'off-formulary' prescribing could occur without restriction unless resources were available to monitor prescribing behaviour closely. National guidelines exist to guide the use of specific products,[4 6 27 28] however, national standards to guide choice across the range of wound care products would reduce variation of product use and guide more rational prescribing.[53]

Engagement in research

Research was raised as a factor influencing wound care in only one, highly research-active provider organisation. In this site, well-established links with university researchers had been highly influential. Current evidence suggests that there is an association between the engagement of individuals and healthcare organisations in research and improvements in healthcare performance.[54] In the other sites, where collaborative links with university researchers were more newly established, research informed decision making was more limited and research generally was viewed with caution. Much of the discussion around acquiring knowledge and skills to inform wound care decisions was related to experiential influences; day-to-day wound

For peer review only

care experience, watching others and consulting with more experienced colleagues and specialists. This finding is in line with other research showing that experiential learning and the social influence of peers rather than research knowledge are major influencers on nursing practices.[55-57]

If evidence obtained from research is to inform management and practice, robust, long-term strategies to support and facilitate its use will be required. In England, the NIHR funded research that incentivises co-production of research e.g., NIHR CLAHRCs represent an on-going nationwide experiment to close the distance between research production and research use.

Limitations

The main limitation is the sample which was taken from community healthcare provider organisations in the North of England and included only one research-active organisation. Inclusion of participants from a larger geographical population may have provided different views, however, we captured many of the issues affecting healthcare (such as work pressures, staff shortages and limited resources) across the UK[30 58 59] and further afield[60 61] due to the financial healthcare crisis worldwide. We would have preferred to include more than one research-active organisation but due to the limited number of research-active organisations within our geographical area as well as funding and time limitations we could not recruit more. We were able to recruit the recommended number of participants for each focus group but work pressures dictated the range of clinical professionals and for one group there were no podiatrists which may have reduced the diversity of views, for that particular interview. However there was good representation from podiatrists across the other groups ranging from senior management to junior positions. Only one research nurse was able to participate and as the research-active organisation was the only organisation to employ a small team of wound research nurses it is not surprising that we could only recruit one.[30 58-61]

A challenge of using the TDF was the overlap across domains such as K*nowledge* and *Skills*, *Beliefs about consequences* and *Social/professional role and identity.* Other authors have reported similar challenges.[17 62] The

For peer review only - http://bmjopen.bmj.com/site/about/guidelines.xhtml

recently published guide to using the TDF, addresses these and other challenges to promote the use of the TDF to a wider audience.[14]

Finally, our aim in this study has been to surface factors that could potentially explain variations in the delivery of wound care. We of course recognise that wound care is complex and multifaceted involving a wide range of behaviours. Given this, we recognise that any formal attempts to develop strategies to modify existing practices and behaviours will require a level of granularity beyond what is available in the data presented. Our study does shed light on those domains where those future efforts should focus.

Conclusions

Our study provides new insight into the role experiential learning and social influences play in determining management and treatment choices and on the limited influence of evidence obtained from research. Workforce pressures and limited resources are perceived by the participants to impede care by reducing patient access to services, the ability to provide holistic care. Co-production of research evidence through participative collaboration between university and healthcare provider organisations may offer a supportive route to addressing issues, implementing sustainable changes to practice and service delivery and a resolute commitment to research use amongst clinical professionals.

Figure Legends

Figure 1. Qualitative analysis using a seven step framework method

Figure 2. Coding Tree showing the four salient domains with connected sub themes

For peer review only - http://bmjopen.bmj.com/site/about/guidelines.xhtml

References

1. Cullum N, Buckley H, Dumville J, et al. Wounds Research for Patient Benefit: a 5 year programme of research. *Programme Grants Appl Res* 2016;4(13)
2. Vowden K. Complex wound or complex patient? Strategies for treatment. *Br J Community Nurs* 2005;Suppl:S6, S8, S10 passim. [published Online First: 2005/06/10]
3. Gray TA, Dumville JC, Christie J, et al. Rapid research and implementation priority setting for wound care uncertainties. *PLoS One* 2017;12(12):e0188958. doi: 10.1371/journal.pone.0188958 [published Online First: 2017/12/06]
4. Coleman S, Nelson EA, Vowden P, et al. Development of a generic wound care assessment minimum data set. *J Tissue Viability* 2017;26(4):226-40. doi: 10.1016/j.jtv.2017.09.007
5. Ashby RL, Gabe R, Ali S, et al. VenUS IV (Venous leg Ulcer Study IV) - compression hosiery compared with compression bandaging in the treatment of venous leg ulcers: a randomised controlled trial, mixed-treatment comparison and decision-analytic model. *Health Technol Assess* 2014;18(57):1-293, v-vi. doi: 10.3310/hta18570
6. NICE. Chronic wounds: advanced wound dressings and antimicrobial dressings: Evidence summary [ESMPB2]. London: National Institute for Health and Care Excellence, 2016.
7. Gray TA, Rhodes S, Atkinson RA, et al. Opportunities for better value wound care: a multiservice, cross-sectional survey of complex wounds and their care in a UK community population. *BMJ Open* 2018;8(3):e019440. doi: 10.1136/bmjopen-2017-019440 [published Online First: 2018/03/25]
8. SIGN. Management of chronic venous leg ulcers: A national clinical quideline (120): Scottish Intercollegiate Guidelines Network, 2010.
9. Ashby RL, Gabe R, Ali S, et al. Clinical and cost-effectiveness of compression hosiery versus compression bandages in treatment of venous leg ulcers (Venous leg Ulcer Study IV, VenUS IV): a randomised controlled trial. *Lancet* 2014;383(9920):871-9. doi: 10.1016/S0140-6736(13)62368-5
10. Adderley U, Evans K, Coleman S. Reducing unwarranted variation in chronic wound care. *Wounds UK* 2017;13(4):22-27.
11. England N. Leading Change, Adding Value: A framework for nursing, Midwifery and care staff London, UK: NHS England; 2016 [Available from: https://www.england.nhs.uk/wp-content/uploads/2016/05/nursing-framework.pdf.
12. Cane J, O'Connor D, Michie S. Validation of the theoretical domains framework for use in behaviour change and implementation research. *Implement Sci* 2012;7:37. doi: 10.1186/1748-5908-7-37 [published Online First: 2012/04/26]
13. Michie S, Johnston M, Abraham C, et al. Making psychological theory useful for implementing evidence based practice: a consensus approach. *Qual Saf Health Care* 2005;14(1):26-33. doi: 10.1136/qshc.2004.011155 [published Online First: 2005/02/05]
14. Atkins L, Francis J, Islam R, et al. A guide to using the Theoretical Domains Framework of behaviour change to investigate implementation problems. *Implement Sci* 2017;12(1):77. doi: 10.1186/s13012-017-0605-9
15. Craig LE, Taylor N, Grimley R, et al. Development of a theory-informed implementation intervention to improve the triage, treatment and transfer of stroke patients in emergency departments using the Theoretical Domains Framework (TDF): the T(3) Trial. *Implement Sci* 2017;12(1):88. doi: 10.1186/s13012-017-0616-6
16. Murphy K, O'Connor DA, Browning CJ, et al. Understanding diagnosis and management of dementia and guideline implementation in general practice: a qualitative study using the theoretical domains framework. *Implement Sci* 2014;9:31. doi: 10.1186/1748-5908-9-31
17. Phillips CJ, Marshall AP, Chaves NJ, et al. Experiences of using the Theoretical Domains Framework across diverse clinical environments: a qualitative study. *Journal of multidisciplinary healthcare* 2015;8:139-46. doi: 10.2147/JMDH.S78458 [published Online First: 2015/04/04]
18. Carlsen B, Glenton C. What about N? A methodological study of sample-size reporting in focus group studies. *BMC Med Res Methodol* 2011;11:26. doi: 10.1186/1471-2288-11-26 [published Online First: 2011/03/15]
19. Guest G, Namey E, McKenna K. How Many Focus Groups Are Enough? Building an Evidence Base for Nonprobability Sample Sizes. *SAGE journals* 2016;29(1):3-22. doi: https://doi.org/10.1177/1525822X16639015
20. Krueger RA, Casey MA. Chapter 4. Participants in a Focus Group. Focus Groups: A Practical Guide for Applied Research. 5 ed. Thousand Oaks, C.A.: Sage Publications Inc 2015:79-101.
21. Tang KC, Davis A. Critical factors in the determination of focus group size. *Fam Pract* 1995;12(4):474-5. [published Online First: 1995/12/01]
22. Noble H, Smith J. Issues of validity and reliability in qualitative research. *Evid Based Nurs* 2015;18(2):34-5. doi: 10.1136/eb-2015-102054 [published Online First: 2015/02/06]
23. Bloor M, Frankland J, Thomas M, et al. Focus Groups in Social Research. London: Sage 2001.

For peer review only

For peer review only - http://bmjopen.bmj.com/site/about/guidelines.xhtml

24. Gale NK, Heath G, Cameron E, et al. Using the framework method for the analysis of qualitative data in multi-disciplinary health research. *BMC Med Res Methodol* 2013;13:117. doi: 10.1186/1471-2288-13-117
25. Ritchie J, Spencer L. Qualitative data analysis for applied policy research. In: Bryman B, Burgess R, eds. Analyzing qualitative data. London and New York: Routledge 1994:173-94.
26. NICE. Developing and updating local formularies [MPG1]. Medicines practice guideline: National Institute for Health ad Care Excellence, 2014.
27. NICE. Pressure ulcers: prevention and management Clinical guideline [CG179]: National Institute for Health and Care Excellence, 2014.
28. NICE. Diabetic foot problems: prevention and management [NG19]: National Institute for Health and Care Excellence, 2015.
29. Ball J, Philippou J, Pike G, et al. Survey of district and community nurses in 2013: report to the Royal College of Nursing. London: Royal College of Nursing, 2014.
30. Maybin J, Charles A, Honeyman m. Understanding quality in district nursing services: Learning from patients, carers and staff. London: The King's Fund, 2016.
31. National Quality Board. Safe, sustainable and productive staffing; An improvement resource for the district nursing service 1ed. London: National Quality Board 2017.
32. Buchan J, Charlesworth A, Gershlick B, et al. Rising pressure: the NHS workforce challenge: Workforce profile and trends of the NHS in England. London: The Health Foundation 2017.
33. OECD. The Nursing Workforce Health Statistics: Organisation for Economic Co-operation and Development 2017.
34. NHS England. Framework for commissioning community nursing. London: NHS England, 2015.
35. Goodwin N, Sonala L, Thiel V, et al. Co-ordinated care for people with complex chronic conditions: Key lessons and markers for success. London: The King's Fund, 2013.
36. HEE. Raising the Bar. Shape of Caring: A Review of the Future Education and Training of Registered Nurses and Care Assistants. London: Health Education England, 2018.
37. NICE. Transition between inpatient hospital settings and community or care home settings for adults with social care needs Nice guideline [NG27] National Institute for Health and Clinical Excellence 2015.
38. Public Health England. Facing the Facts, Shaping the Future: A draft health and care workforce strategy for England to 2027. London: Public Health England, 2017.
39. Vimalananda VG, Gupte G, Seraj SM, et al. Electronic consultations (e-consults) to improve access to specialty care: a systematic review and narrative synthesis. *J Telemed Telecare* 2015;21(6):323-30. doi: 10.1177/1357633X15582108 [published Online First: 2015/05/23]
40. Madden M. Alienating evidence based medicine vs. innovative medical device marketing: a report on the evidence debate at a Wounds conference. *Soc Sci Med* 2012;74(12):2046-52. doi: 10.1016/j.socscimed.2012.02.026
41. Greatbatch. A false economy: Cuts to Continuing Professional Development funding for nursing, midwifery and the Allied Health Professions in England London: Council of Deans of Health, 2016.
42. Ousey K. Reducing funding for CPD courses: what does it mean for tissue viability? . *Wounds UK* 2016;13(1):10-15.
43. Frenk J, Chen L, Bhutta ZA, et al. Health professionals for a new century: transforming education to strengthen health systems in an interdependent world. *Lancet* 2010;376(9756):1923-58. doi: 10.1016/S0140-6736(10)61854-5 [published Online First: 2010/11/30]
44. Rafferty AM, Xyrichis A, Caldwell C. Post-graduate education and career pathways in nursing: a policy brief London: King's College London., 2015.
45. Thompson C, Stapley S. Do educational interventions improve nurses' clinical decision making and judgement? A systematic review. *Int J Nurs Stud* 2011;48(7):881-93. doi: 10.1016/j.ijnurstu.2010.12.005
46. Werdin F, Tennenhaus M, Schaller HE, et al. Evidence-based management strategies for treatment of chronic wounds. *Eplasty* 2009;9:e19.
47. Dumville JC, Deshpande S, O'Meara S, et al. Foam dressings for healing diabetic foot ulcers. *Cochrane Database Syst Rev* 2013(6):CD009111. doi: 10.1002/14651858.CD009111.pub3
48. Dumville JC, O'Meara S, Deshpande S, et al. Hydrogel dressings for healing diabetic foot ulcers. *Cochrane Database Syst Rev* 2013(7):CD009101. doi: 10.1002/14651858.CD009101.pub3
49. Dumville JC, Stubbs N, Keogh SJ, et al. Hydrogel dressings for treating pressure ulcers. *Cochrane Database Syst Rev* 2015(2):CD011226. doi: 10.1002/14651858.CD011226.pub2
50. Forster R, Pagnamenta F. Dressings and topical agents for arterial leg ulcers. *Cochrane Database Syst Rev* 2015(6):CD001836. doi: 10.1002/14651858.CD001836.pub3
51. Jull AB, Cullum N, Dumville JC, et al. Honey as a topical treatment for wounds. *Cochrane Database Syst Rev* 2015(3):CD005083. doi: 10.1002/14651858.CD005083.pub4
52. O'Meara S, Martyn-St James M. Foam dressings for venous leg ulcers. *Cochrane Database Syst Rev* 2013(5):CD009907. doi: 10.1002/14651858.CD009907.pub2

For peer review only - http://bmjopen.bmj.com/site/about/guidelines.xhtml

53. Health Service Executive. National best practice and evidence based guidelines for wound managment. Dublin, Ireland: Health Service Executive, 2009.

54. Boaz A, Hanney S, Jones T, et al. Does the engagement of clinicians and organisations in research improve healthcare performance: a three-stage review. *BMJ Open* 2015;5(12):e009415. doi: 10.1136/bmjopen-2015-009415 [published Online First: 2015/12/15]

55. Luker KA, Kenrick M. An exploratory study of the sources of influence on the clinical decisions of community nurses. *J Adv Nurs* 1992;17(4):457-66.

56. McCaughan D, Thompson C, Cullum N, et al. Nurse practitioner and practice nurses' use of research information in clinical decision making: findings from an exploratory study. *Fam Pract* 2005;22(5):490-7. doi: 10.1093/fampra/cmi042

57. O'Leary D F, Mhaolrunaigh SN. Information-seeking behaviour of nurses: where is information sought and what processes are followed? *J Adv Nurs* 2012;68(2):379-90. doi: 10.1111/j.1365-2648.2011.05750.x

58. Robertson R, Wenzel L, Thompson J, et al. Understanding NHS financial pressures How are they affecting patient care? London: The King's Fund, 2017.

59. Jackson C. Implementing a District and Community Nursing workload tool, to determine safe staffing levels and skill mix in a community care provider organisation: An economic assessment of potential benefits for workforce planning. Kent: Canterbury Christ Church University 2016.

60. Saltman RB. The impact of slow economic growth on health sector reform: a cross-national perspective. *Health Econ Policy Law* 2018:1-24. doi: 10.1017/S1744133117000445

61. Karanikolos M, Mladovsky P, Cylus J, et al. Financial crisis, austerity, and health in Europe. *Lancet* 2013;381(9874):1323-31. doi: 10.1016/S0140-6736(13)60102-6

62. Bussieres AE, Patey AM, Francis JJ, et al. Identifying factors likely to influence compliance with diagnostic imaging guideline recommendations for spine disorders among chiropractors in North America: a focus group study using the Theoretical Domains Framework. *Implement Sci* 2012;7:82. doi: 10.1186/1748-5908-7-82

For peer review only

For peer review only - http://bmjopen.bmj.com/site/about/guidelines.xhtml

Funding

This project was funded by the National Institute for Health Research Collaboration for Leadership in Applied Health Research and Care (NIHR CLAHRC) Greater Manchester. The NIHR CLAHRC Greater Manchester is a partnership between providers and commissioners from the NHS, industry and the third sector, as well as clinical and research staff from the University of Manchester. The views expressed in this article are those of the authors and not necessarily those of the NHS, NIHR or the Department of Health.

Contributors

NC, JD and TG conceived the idea and design for the overall project, PW contributed to further development of the study design. TG, PW and JD collected the data. TG and PW were responsible for data analyses. TG created the original draft of the manuscript. All authors contributed to the interpretation of study findings, critical revision of the manuscript for important intellectual content and approval of the final manuscript.

Acknowledgements

The authors are grateful to many colleagues within the NIHR CLAHRC Greater Manchester Wound Care Programme, who have assisted with and supported this work. We thank all participants for giving their time, sharing their views and for their enthusiasm throughout

Conflicts of interest

None declared

Data sharing statement

Requests for access to data should be addressed to the corresponding author.

For peer review only

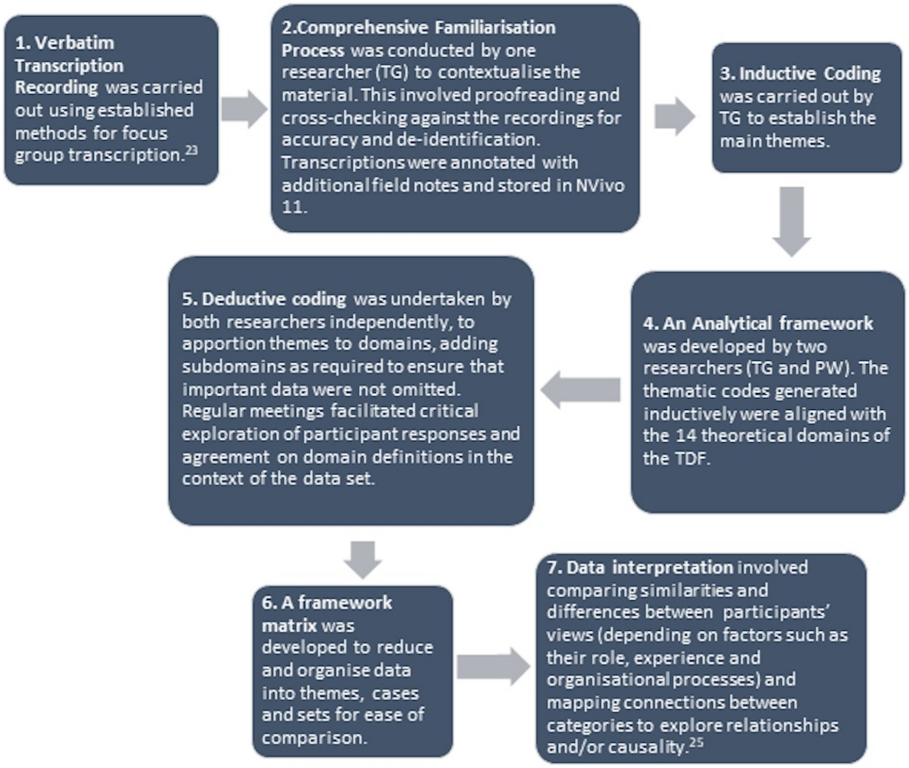

Figure 1. Qualitative analysis using a seven step framework method

90x90mm (300 x 300 DPI)

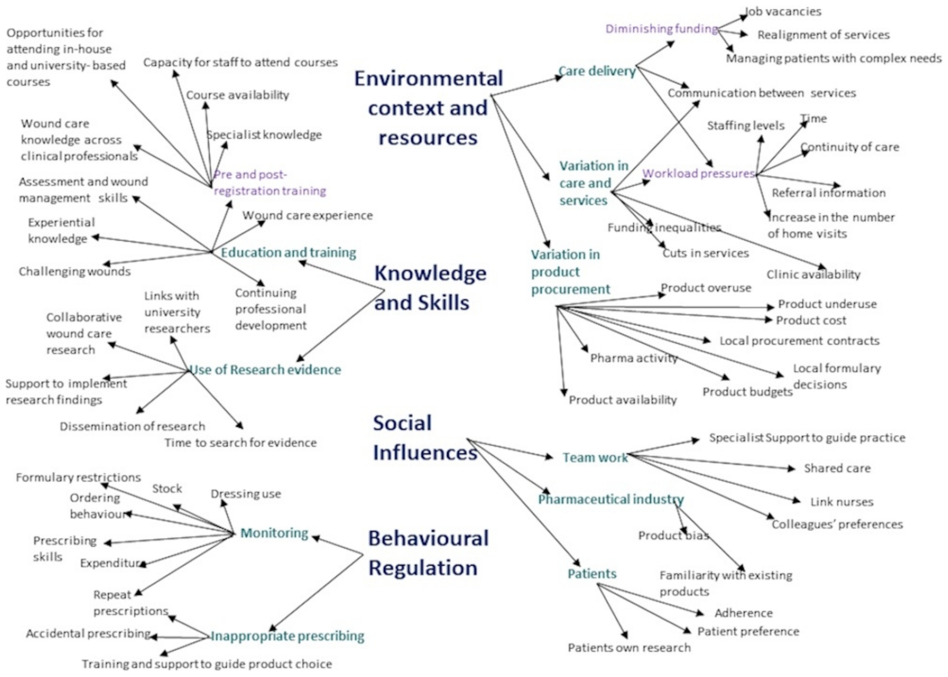

Figure 2. Coding Tree showing the four salient domains with connected sub themes

89x89mm (300 x 300 DPI)

The University of Manchester

National Institute for Health Research

Appendix 1: What Factors Influence Community Wound Care: Clinical Professional Focus Group Questions

Questions	Prompts for further exploration	TDF Domains
How do you decide which dressing or treatment to use for which patient?	What factors contribute and how is it/are they obtained? Please provide examples to explain answers<br>• Knowledge and skills,<br>○ Under/post grad training/regular updates<br>○ Peers/networking<br>○ Experience/Expertise<br>○ Preferences<br>○ Specialist support<br>○ Conferences/seminars<br>○ Pharmaceutical reps/fact sheets<br>• Research evidence<br>○ Reading journals<br>○ On-line search<br>○ National guidelines<br>○ Communicated via wound care specialists<br>• Patient and carers' influence<br>○ Lifestyle<br>○ Adherence<br>○ Choice/Preference<br>○ Anatomical factors /dressing suitability for foot in mobile patient	Skills Knowledge<br><br>Social professional role and identity<br><br>Behavioral Regulation<br><br>Environmental context and resources<br><br>Beliefs about capabilities<br><br>Memory, attention and decision processes<br><br>Beliefs about consequences<br><br>Motivation and goals<br><br>Emotion
Are there any environmental (organisational or resource based) factors that influence your prescribing practice?	Are the following enablers or barriers?<br>• Processes e.g. having a formulary in place?<br>○ If a formulary is in place is ordering from it mandatory?<br>• Product cost?<br>• Value for money?<br>○ Are some products worth paying more for e.g. silver/soft silicon?<br>○ What additional benefits do they provide?<br>○ How do you justify the additional cost?<br>• Product availability?<br>• Product knowledge? Why choose one product over another?r<br>• Memory (considering the number of products available)?<br>• Training? Competence?<br>• Caseload? Automony?<br>• Team support?	Behavioral Regulation<br><br>Environmental context and resources<br><br>Knowledge<br><br>Skills<br><br>Beliefs about capabilities<br><br>Memory, attention and decision processes<br><br>Beliefs about capabilities

The University of Manchester

National Institute for Health Research

Questions	Prompts for further exploration	TDF Domains
Do you have any influence on what products are included in your Trust formulary?	How?<br><br>Who else is involved?<br><br>If not you – who decides	Social professional role and identity<br><br>Skills<br><br>Environmental context and resources<br><br>Beliefs about capabilities<br><br>Nature of the behaviors
Have other people or situations ever caused you to change your wound care practices?	An incident? What happened?<br><br>Service reconfiguration? Why was this necessary?<br><br>A change in policy? Why was the policy changed? How was the change implemented?<br><br>A colleague? An expert in the field?<br><br>What processes are in place to share practice relating to product usage?<br><br>Do current networks adequately promote shared cared between teams and services? If not what do you think needs to be done to improve this?	Behavioral Regulation<br><br>Environmental context and resources<br><br>Nature of the behaviors<br><br>Social influences
How do you know you are/your service or your trust is making the right decisions regarding product use and service delivery?	Have you completed any audits of clinical care, clinical outcomes or service outcomes?<br>• Are prescribing practices audited? If so how frequently?<br>• Are prescribing skills audited? If so how frequently?<br>• Are prescribing skills regularly monitored/ appraised? If so how frequently?<br>• Do you have a PDP? Do attend regular personal development reviews? How often?<br>• Do you receive regular updates at trust or service level regarding service delivery achievements?<br>What other measures are in place to monitor prescribing practices?	Behavioral Regulation<br><br>Motivation and goals

For peer review only

The University of Manchester

National Institute for Health Research

Now you have had time to look at the wound care product expenditure………..

Questions	Prompts for further exploration	TDF Domains
Are the overall figures what you were expecting?	Is the overall spend higher or lower than you were expecting?<br><br>What do you think has caused the difference (if any)?<br><br>How do you feel about the differences or similarities with neighboring Trusts and national figures?	Knowledge<br><br>Beliefs about consequences<br><br>Behavioral Regulation<br><br>Environmental context and resources
Does the expenditure for any particular product group surprise you?	Are you surprised that it is low or high expenditure?<br><br>Why does it surprise you?<br><br>What do you think has caused this?<br><br>How do you feel about the differences with neighboring Trusts and national figures?	Knowledge<br><br>Beliefs about consequences<br><br>Environmental context and resources<br><br>Behavioral Regulation
Do you think there is over or under use of any product group?	Which group(s)? Is this over or under used?<br><br>What do you think has caused this?<br><br>Prior to seeing the figures, which product groups did you believe were used the most frequently?<br><br>Prior to seeing the figures, which product groups did you believe were used the least frequently?<br><br>How do you feel about the comparison with neighboring Trusts and national figures?	Knowledge<br><br>Skills<br><br>Beliefs about consequences<br><br>Environmental context and resources

For peer review only

The University of Manchester

National Institute for Health Research

Appendix 2. What Factors Influence Community Wound Care: Non-clinical Professional Focus Group Questions

Introductory statement to clarify the focus: As you all represent different organisations and have different roles, our questions will aim to establish the procurement processes and practices across four community healthcare organisations (that have partnered with CLAHRC GM to deliver this work) and neighbouring partner CCGs. When we say 'local' we are referring to the 14 services involved. We will use prompts to explore each question further so that we have a clear understanding of each of the services involved.

Questions	Prompts for further exploration	TDF Domains
What procurement processes are in place locally?	Stock list only? Prescribing only? Combination?<br><br>How is information about the systems/practices circulated to community and primary care staff? Are communication channels monitored?<br><br>Do Trusts/CCGs have a formulary?<br>• How are they compiled/updated?<br>• Who is involved?<br>• Who influences what is included?<br><br>What are the drivers for changing processes/practice? Do you have examples?<br>• Incidents?<br>• Audit findings?<br>• Service reconfiguration?<br>• Research evidence?<br>• Lessons learnt from other services?	Environmental context and resources<br><br>Knowledge<br><br>Skills<br><br>Social professional role and identity<br><br>Social influences

For peer review only

The University of Manchester

National Institute for Health Research

Questions	Prompts for further exploration	TDF Domains
Are there any factors that influence procurement decisions?	Are the following enablers or barriers?<br>• Product cost?<br>• Product availability?<br>• Product knowledge?<br>• Memory (considering the number of products available)?<br>• Training?<br>• Competence?<br>• Team support?<br>• Company reps<br>○ access monitored/unmonitored<br>○ Incentives	Behavioral Regulation<br><br>Environmental context and resources<br><br>Knowledge<br><br>Skills<br><br>Beliefs about capabilities<br><br>Memory, attention and decision processes
What locally agreed CQUINs/policies are in place for wound care management?	What do these entail?<br><br>Are there any requirements to conduct audits to monitor adherence to agreed wound care management pathway?<br>• Six monthly/yearly?<br>• What are the consequences for poor adherence<br><br>Do they include incentive schemes?<br>Does they include a commitments to reduce spend e.g. reduce silver dressings spend.<br>Do these include a commitment to undertake an ongoing programme of educational training to community or primary care staff on agreed wound management pathway (inc formulary)?<br><br>Do they include a commitment to benchmark with other organisations? (Which?)	Behavioral Regulation<br><br>Environmental context and resources<br><br>Beliefs about consequences<br><br>Motivation and goals<br>Skills
Do healthcare professionals follow policy when ordering /prescribing wound care products?	What percentage of products are ordered/prescribed 'off formulary'<br><br>Are there specific products that are prescribed regularly 'off formulary'?<br><br>Are there any incentives to promote good prescribing/ordering practices?<br><br>If a stock list exists, are stock products sometimes prescribed rather than ordered from the stock list?<br><br>What are the consequences of not following policy/ordering off formulary?<br><br>What do you think can be done to improve adherence to policy?	Behavioral Regulation<br><br>Environmental context and resources<br><br>Beliefs about consequences

The University of Manchester

National Institute for Health Research

Now you have had time to look at the wound care product expenditure………..		
**Questions**	**Prompts for further exploration**	**TDF Domains**
How do you feel about the differences or similarities in expenditure locally compared to the national spend?	Is the overall spend higher or lower than you would like it to be?<br><br>How do you feel about the differences or similarities with the rest of GM and the national figures?<br><br>If overall spend is higher than average what do you think can be done to reduce the spend?	Knowledge<br><br>Beliefs about consequences<br><br>Behavioral Regulation<br><br>Environmental context and resources
Is the expenditure for any particular product higher or lower than you think it should be?	Why do to think expenditure is higher or lower?<br><br>Which type of HCP is contributing to the high/low expenditure?<br><br>How does this compares with the rest of the region and the national figures?	Knowledge<br><br>Beliefs about consequences<br><br>Environmental context and resources<br><br>Behavioral Regulation
Do you think there is over or under use of any product group?	Which group(s)? Is this over or under use?<br><br>What do you think has caused this?<br><br>How does this compare with regional and the national figures?	Knowledge<br><br>Skills<br><br>Beliefs about consequences<br><br>Environmental context and resources

For peer review only

Item No	Topic	Guide Questions/Descriptions	Reported on Page No (clean copy)
**Domain 1 Research Team and reflexivity**			
Personal Characteristics			
1	Interviewer/facilitator	Which author/s conducted the interview or focus group?	8
2	Credentials	What were the researcher's Credentials E.g. PhD, MD Occupation	8
3	Occupation	What was their occupation at the time of the study?	8
4	Gender	Was the researcher male or female?	8
5	Experience and training	What experience or training did the researcher have?	8
6	Relationship established	Was a relationship established prior to study commencement?	7
7	Participant knowledge of the interviewer	What did the participants know about the researcher? e.g. personal goals, reasons for doing the research	7
8	Interviewer characteristics	What characteristics were reported about the interviewer/facilitator? e.g. Bias, assumptions, reasons and interests in the research topic	8
**Domain 2: Study design**			
Theoretical framework			
9	Methodological orientation and Theory	What methodological orientation was stated to underpin the study? e.g. grounded theory, discourse analysis, ethnography, phenomenology, content analysis	5/9
10	Sampling	How were participants selected? e.g. purposive, convenience, consecutive, snowball	7
11	Method of approach	How were participants approached? e.g. face-to-face, telephone, mail, email	7
12	Sample size	How many participants were in the study?	7/9/10
13	Non-participation	How many people refused to participate or dropped out? Reasons?	9
	Setting		
14	Setting of data collection	Where was the data collected? e.g. home, clinic, workplace	7
15	Presence of nonparticipants	Was anyone else present besides the participants and researchers?	8
16	Description of sample	What are the important characteristics of the sample? e.g. demographic data, date	10
	Data collection		
	Interview guide	Were questions, prompts, guides provided by the authors? Was it pilot tested?	Appendix 1and 2/8
	Repeat interviews	Were repeat inter views carried out? If yes, how many?	N/A
	Audio/visual recording	Did the research use audio or visual recording to collect the data?	8
	Field notes	Were field notes made during and/or after the interview or focus group?	8

For peer review only

	Duration	What was the duration of the inter views or focus group?	9
	Data saturation	Was data saturation discussed?	7
	Transcripts returned	Were transcripts returned to participants for comment and/or correction?	8
**Domain 3: analysis and findings**			
	Data analysis		
	Number of data coders	How many data coders coded the data?	Figure 1
	Description of the coding tree	Did authors provide a description of the coding tree?	Figure 2
	Derivation of themes	Were themes identified in advance or derived from the data?	8/Figure 1
	Software	What software, if applicable, was used to manage the data?	9/Figure 1
	Participant checking	Did participants provide feedback on the findings?	8
	Reporting		
	Quotations presented	Were participant quotations presented to illustrate the themes/findings? Was each quotation identified? e.g. participant number	11-15
	Data and findings consistent	Was there consistency between the data presented and the findings?	10-15
	Clarity of major themes	Were major themes clearly presented in the findings?	10-15
	Clarity of minor themes	Is there a description of diverse cases or discussion of minor themes?	10

Developed from: Tong A, Sainsbury P, Craig J. Consolidated criteria for reporting qualitative research (COREQ): a 32-item checklist for interviews and focus groups. International Journal for Quality in Health Care. 2007. Volume 19, Number 6: pp. 349 – 357

For peer review only
